# Global patterns of mortality in international migrants: a systematic review and meta-analysis

**DOI:** 10.1016/S0140-6736(18)32781-8

**Published:** 2018-12-15

**Authors:** Robert W Aldridge, Laura B Nellums, Sean Bartlett, Anna Louise Barr, Parth Patel, Rachel Burns, Sally Hargreaves, J Jaime Miranda, Stephen Tollman, Jon S Friedland, Ibrahim Abubakar

**Affiliations:** a Centre for Public Health Data Science, Institute of Health Informatics, University College London, London, UK; b Institute for Global Health, University College London, London, UK; c Institute of Infection and Immunity, St George's, University of London, London, UK; d International Health Unit, Section of Infectious Diseases, Imperial College London, London, UK; e Nuffield Department of Medicine, University of Oxford, Oxford, UK; f Department of Medicine, University of Cambridge, Cambridge, UK; g School of Public Health, Imperial College London, London, UK; h CRONICAS Center of Excellence in Chronic Diseases and Department of Medicine, School of Medicine, Universidad Peruana Cayetano Heredia, Lima, Peru; i MRC/Wits Rural Public Health and Health Transitions Research Unit, School of Public Health, University of the Witwatersrand, Johannesburg, South Africa; j INDEPTH Network, Accra, Ghana

## Abstract

**Background:**

258 million people reside outside their country of birth; however, to date no global systematic reviews or meta-analyses of mortality data for these international migrants have been done. We aimed to review and synthesise available mortality data on international migrants.

**Methods:**

In this systematic review and meta-analysis, we searched MEDLINE, Embase, the Cochrane Library, and Google Scholar databases for observational studies, systematic reviews, and randomised controlled trials published between Jan 1, 2001, and March 31, 2017, without language restrictions. We included studies reporting mortality outcomes for international migrants of any age residing outside their country of birth. Studies that recruited participants exclusively from intensive care or high dependency hospital units, with an existing health condition or status, or a particular health exposure were excluded. We also excluded studies limited to maternal or perinatal outcomes. We screened studies using systematic review software and extracted data from published reports. The main outcomes were all-cause and International Classification of Diseases, tenth revision (ICD-10) cause-specific standardised mortality ratios (SMRs) and absolute mortality rates. We calculated summary estimates using random-effects models. This study is registered with PROSPERO, number CRD42017073608.

**Findings:**

Of the 12 480 articles identified by our search, 96 studies were eligible for inclusion. The studies were geographically diverse and included data from all global regions and for 92 countries. 5464 mortality estimates for more than 15·2 million migrants were included, of which 5327 (97%) were from high-income countries, 115 (2%) were from middle-income countries, and 22 (<1%) were from low-income countries. Few studies included mortality estimates for refugees (110 estimates), asylum seekers (144 estimates), or labour migrants (six estimates). The summary estimate of all-cause SMR for international migrants was lower than one when compared with the general population in destination countries (0·70 [95% CI 0·65–0·76]; *I*^2^=99·8%). All-cause SMR was lower in both male migrants (0·72 [0·63–0·81]; *I*^2^=99·8%) and female migrants (0·75 [0·67–0·84]; *I*^2^=99·8%) compared with the general population. A mortality advantage was evident for refugees (SMR 0·50 [0·46–0·54]; *I*^2^=89·8%), but not for asylum seekers (1·05 [0·89–1·24]; *I*^2^=54·4%), although limited data was available on these groups. SMRs for all causes of death were lower in migrants compared with the general populations in the destination country across all 13 ICD-10 categories analysed, with the exception of infectious diseases and external causes. Heterogeneity was high across the majority of analyses. Point estimates of all-cause age-standardised mortality in migrants ranged from 420 to 874 per 100 000 population.

**Interpretation:**

Our study showed that international migrants have a mortality advantage compared with general populations, and that this advantage persisted across the majority of ICD-10 disease categories. The mortality advantage identified will be representative of international migrants in high-income countries who are studying, working, or have joined family members in these countries. However, our results might not reflect the health outcomes of more marginalised groups in low-income and middle-income countries because little data were available for these groups, highlighting an important gap in existing research. Our results present an opportunity to reframe the public discourse on international migration and health in high-income countries.

**Funding:**

Wellcome Trust, National Institute for Health Research, Medical Research Council, Alliance for Health Policy and Systems Research, Department for International Development, Fogarty International Center, Grand Challenges Canada, International Development Research Centre Canada, Inter-American Institute for Global Change Research, National Cancer Institute, National Heart, Lung and Blood Institute, National Institute of Mental Health, Swiss National Science Foundation, World Diabetes Foundation, UK National Institute for Health Research Imperial Biomedical Research Centre, Imperial College Healthcare Charity, and European Society for Clinical Microbiology and Infectious Diseases (ESCMID) Study Group Research Funding for the ESCMID Study Group for Infections in Travellers and Migrants.

**Research in context**

**Evidence before this study**

More than 258 million people live outside their country of birth, but little global evidence is available on the mortality of international migrants compared with general populations. In preparation for this analysis, we did a rapid review of PubMed and Google Scholar for studies on mortality in migrant populations. One large analysis of mortality data from six European countries highlighted inconsistencies in the patterns of mortality in migrants, and found variations in mortality across migrant populations whereby all-cause mortality was lower for migrants from east Asia and Latin America, and higher in those migrating from north Africa and eastern Europe compared with general populations. A 2003 review found that all-cause and cause-specific mortality varied across international migrants by country of origin, destination, and migration trajectory. This review did not include a meta-analysis of available data, or present results across International Classification of Diseases-10 categories, and thus was limited in its ability to compare mortality among migrants and the general populations and by major disease categories.

**Added value of this study**

Our systematic review of the published literature provides a robust analysis of the available evidence base, suggesting that overall, mortality among international migrants was lower than the general population in high-income destination countries. Our results might not be generalisable to more marginalised migrants, and in particular forced migrants and those living in low-income and middle-income countries, as a result of the scarcity of data on their mortality outcomes. Therefore, caution is urged when generalising estimates to these populations and locations. International migrants had increased mortality due to infectious diseases (viral hepatitis, tuberculosis, and HIV) and external causes of deaths (assault and events of undetermined intent), and no mortality advantage was identified for asylum seekers, for whom limited data were available. Our systematic review and meta-analysis supports the healthy migrant hypothesis, and provides the most comprehensive synthesis of evidence to date on mortality outcomes in international migrants. Our results also indicate infectious disease and external causes of mortality as two key areas in which opportunities exist for prevention and ability to improve the health of migrants and the wider public. Our results also highlight the need to improve data collection in migrant groups such as refugees, asylum seekers, and undocumented migrants, and migrants living in low-income and middle-income countries, who might be at increased risk of morbidity and mortality and were more likely to be underrepresented. Heterogeneity was high in our study and this could not be explained by subgroup analyses. This heterogeneity indicates that some groups of migrants will continue to have unmet health needs and the summary mortality advantage presented must not be used as a justification for any restrictions in access to health care for migrant groups, which is a growing issue across many countries.

**Implications of all the available evidence**

Public perception that migrants place an undue burden on societies is guiding governments across the globe to tighten access to health care and generate so-called hostile environments for these groups. Our findings contradict claims that migrants are a health burden in high-income countries and suggest that these policies do not align with the available evidence. Our results show that current perceptions underestimate the positive contributions of migrants to host societies in these settings. Migrants have a mortality advantage compared with general populations across the majority of disease categories, with the exception of infectious diseases and external causes. Improving access to health services and treating infectious diseases in specific migrant subpopulations is likely to have multiple benefits: lowering mortality rates, strengthening global health security and effective infection prevention and control, and reducing the burden of diseases such as hepatitis, tuberculosis, and HIV in destination countries. This will require host health services to better adapt to increase their accessibility and responsiveness to the needs of some migrant groups. Risk of mortality from infectious disease varies greatly among migrants and therefore screening should be context and epidemiology specific, codeveloped with migrants, and only done in areas where a health benefit to migrants can be demonstrated. Future research should seek to address the scarcity of data specific to migrant subgroups who are marginalised, in particular forced migrants and those originating from and living in low-income and middle-income countries. These groups might have a higher mortality burden and further research into these populations should be prioritised.

## Introduction

258 million people live outside their country of birth.^1^ These international migrants account for more than 3% of the world's population and originate from diverse backgrounds with health determinants that vary considerably between the countries of origin and destination.

International migrants encompass a range of different subgroups including those who have chosen to migrate (eg, economic, student, and family reunion migrants), and those who might have been forced to migrate due to conflict, persecution, or environmental disasters (eg, refugees, asylum seekers, and undocumented migrants) who are often referred to as forced migrants.

Conflicting evidence exists about the health outcomes of international migrants. Some data^2^ support the healthy migrant hypothesis—an empirically observed mortality advantage among migrants from certain countries of origin, relative to the majority population in host countries^2^—whereas other research^3^, ^4^ shows poorer outcomes in certain disease categories and key migrant groups. Although a mortality advantage in international migrants has been supported by several studies,^5^, ^6^, ^7^, ^8^ evidence^9^ from six European countries suggested that mortality patterns vary by birthplace, with migrants from east Asia and Latin America having lower all-cause mortality and those from Eastern Europe and Africa having higher mortality than the general populations of host countries. Contradictory evidence exists about certain migrant subgroups such as refugees with studies showing both improved,^10^, ^11^ no difference^12^ and worsened^13^ mortality outcomes across a diversity of countries and refugee settings.

Our study aimed to systematically identify and synthesise original research to investigate global patterns of mortality in international migrants. First, we aimed to investigate the relative and absolute mortality (all-cause mortality and International Classification of Diseases, tenth revision [ICD-10] classified cause-specific mortality) in international migrants compared with the general population. Second, we aimed to examine differences in all-cause and ICD-10 classified cause-specific mortality by sex, migrant subgroup (eg, refugee, asylum seeker, student, economic migrant) and region of origin. Third, we aimed to assess how representative the research evidence base was with regard to the mortality of migrants by investigating whether an association exists between cause-specific risk of mortality and the number of studies done by cause-specific ICD-10 disease category.

## Methods

### Search strategy and selection criteria

For this systematic review and meta-analysis, we searched MEDLINE, Embase, the Cochrane Library, and Google Scholar databases for studies published between Jan 1, 2001, and March 31, 2017, reporting mortality in international migrants, without language restrictions. Full search terms are provided in the appendix. We chose to search for studies published after Jan 1, 2001, because a previous systematic review of mortality in migrants had been published by this date, but it did not contain a meta-analysis and did not assess outcomes across all ICD-10 categories.^3^

On Sept 3, 2018, we updated our search using the same databases, search terms, and inclusion criteria.

We included observational (cohort and cross-sectional), systematic reviews, and randomised controlled trials reporting quantitative data on mortality in international migrants of any age residing outside their country of birth.

We excluded studies that recruited participants exclusively from intensive care or high dependency hospital units, with an existing health condition or status (eg, myocardial infarction, HIV, tuberculosis, pregnancy), or a particular health exposure (eg, smoking, high blood pressure). We also excluded studies limited to maternal or perinatal outcomes. The study with the largest or most representative sample was included, and when these were equal, the most recent study was included. Discrepancies in the inclusion or exclusion of papers during screening were discussed until consensus was achieved, and RWA resolved any final discrepancies. This study was done in accordance with the Preferred Reporting Items for Systematic Reviews and Meta-Analysis (PRISMA)^14^ guidelines. The study protocol is available online. Deviations from the protocol are reported in the appendix.

### Data analysis

Five reviewers (RWA, SB, ALB, LBN, and PP) screened titles, abstracts, and full texts using Covidence systematic review software. Two reviewers independently examined citations at each stage. We adapted a previously used data extraction form,^15^ to record study design, year or years of study, country, country of origin, number of participants, standardised mortality ratios (SMRs), absolute mortality rates, and summary descriptions of the study population. Extracted data were reviewed and checked by a second author before cleaning and analysis. Duplicate data were removed for studies reporting information from the same migrant group (by country of destination) for the same mortality outcome and time period.

Outcomes of interest were all-cause and ICD-10 cause-specific SMRs and absolute mortality rates. The number of datapoints that presented cause-specific mortality according to ICD-10 groups was also calculated. We report data by ICD-10 disease category, and converted outcomes from studies reporting data using older ICD versions as necessary.

Four reviewers (SB, ALB, RB, and PP) assessed the risk of bias of included papers using a piloted quality assessment form adapted from the Newcastle Ottawa Scale.^16^ A randomly selected sample (10%) of these assessments was corroborated by LBN.

We used the metafor package (version 2.0) in the statistical software R (version 3.5.1) and random-effects models to calculate pooled estimates of mortality and corresponding 95% CIs. Heterogeneity was assessed using the *I*^2^ statistic, and assessed further in subgroup analyses wherever possible. Mortality point estimates were included in each model with corresponding SEs extracted directly or calculated using CIs for each point estimate.

Subgroup analyses were done when appropriate to assess mortality by sex, migrant type (eg, refugee or asylum seeker), World Bank geographical region of origin, World Bank income level of countries of origin, and evidence quality. The study is registered with PROSPERO, number CRD42017073608.

### Role of the funding source

The funders of the study had no role in study design, data collection, data analysis, data interpretation, writing of the report, or the decision to submit the paper for publication. All authors had full access to all data in the study and had final responsibility for the decision to submit for publication.

## Results

**Figure 1:**
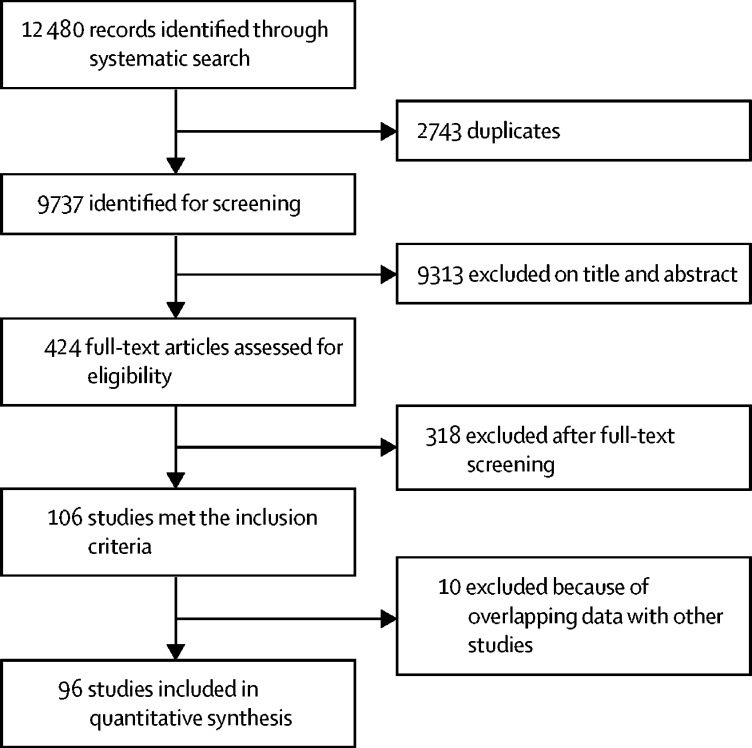
Study selection

We identified 12 480 articles, of which 2743 were duplicates (figure 1). 424 full-text articles were assessed for eligibility (eight studies could not be located), of which 106 met the inclusion criteria and 6283 datapoints were extracted. After the removal of duplicate data, 96 studies and 5464 mortality estimates for more than 15·2 million migrants were included in our analyses. References for all included studies are listed in the appendix. These mortality estimates included 110 estimates for refugees, 144 estimates for asylum seekers, and six estimates for labour migrants. Migrant subgroup could not be determined for 5204 mortality estimates from 88 included studies, with reporting generally referring to these groups as foreign-born rather than specific migrant groups (eg, students or labour migrants).

We identified World Bank geographical region of origin for 4085 (75%) of 5464 mortality estimates, and country of origin for 1720 (31%) of 5464 estimates. International migrants originated from 92 countries and 25 migrant destination countries were included (appendix). Data from the UN Department of Economic and Social Affairs^1^ indicate that in 2017, 165 (64%) of 258 million international migrants lived in high-income countries, with 81 (31%) of 258 million residing in middle-income countries and 11 (5%) of 258 million residing in low-income countries. Of 5464 mortality estimates, 5327 (97%) estimates were from high-income countries, 115 (2%) were from middle-income countries, and 22 (<1%) were from low-income countries.

**Figure 2:**
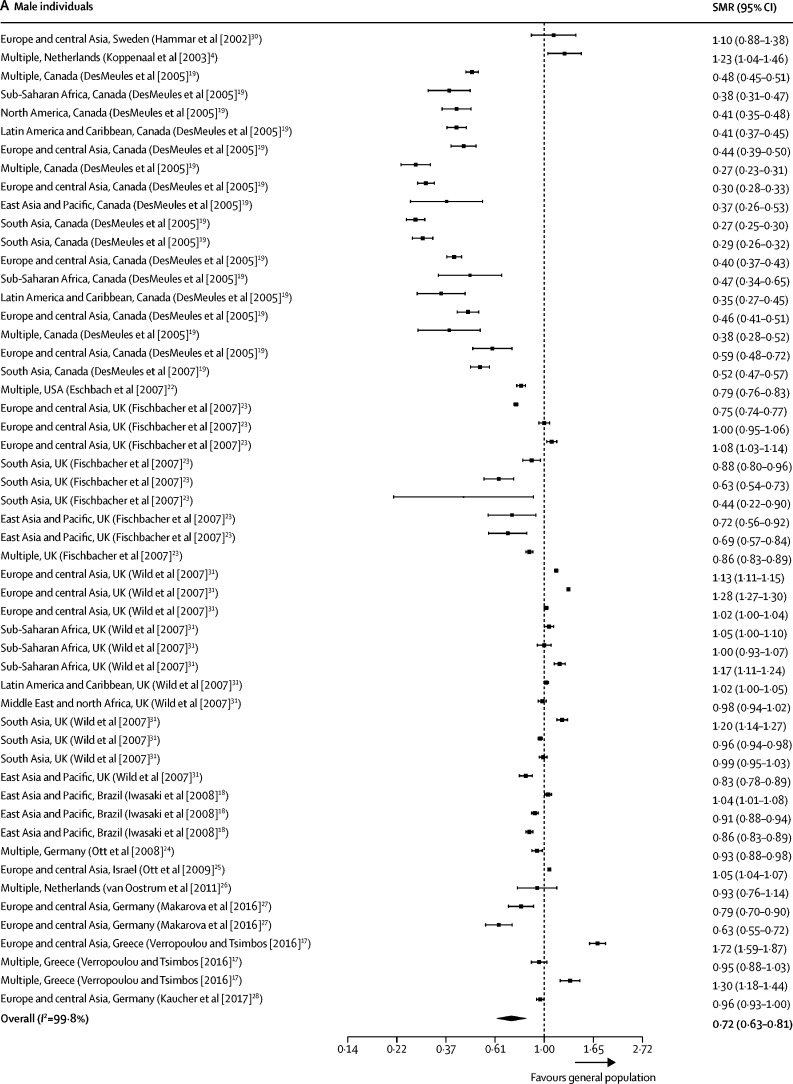

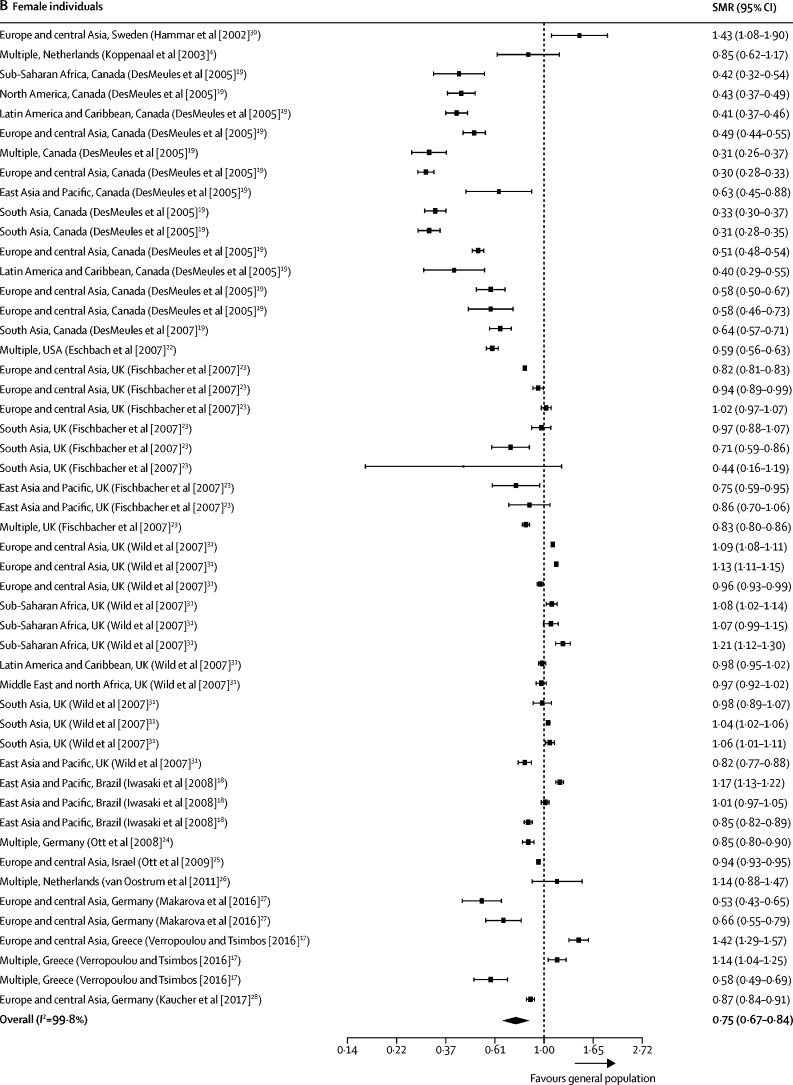
Forest plots of SMRs for all-cause mortality Studies are presented as region of origin, country of study (study [year]). Data are presented for male individuals (A) and female individuals (B). 13 studies reported all-cause mortality estimates, of which 53 estimates were available for men, and 50 were available for women. SMR=standardised mortality ratio.

Our all-cause SMR meta-analyses included data from 16 studies^4^, ^17^, ^18^, ^19^, ^20^, ^21^, ^22^, ^23^, ^24^, ^25^, ^26^, ^27^, ^28^, ^29^, ^30^, ^31^ and 119 mortality estimates (table). Seven studies^17^, ^19^, ^20^, ^23^, ^24^, ^25^ were done using national census data, and therefore included all international migrants reported in these countries. 89 (75%) of 119 included SMR all-cause mortality estimates were less than 1, indicating a migrant mortality advantage. Summary estimates of all-cause SMRs for international migrants were less than 1 when compared with the general population of the host country (0·70 [95% CI 0·65–0·76]; *I*^2^=99·8%; appendix). All-cause SMRs remained less than 1 when analysed by sex (0·72, 0·63–0·81; *I*^2^=99·8%] for men; 0·75 [0·67–0·84]; *I*^2^=99·8% for women; figure 2). Of the 16 studies included in the all-cause meta-analyses, all were done in high-income settings with the exception of one study^18^ from Brazil.

**Table:** Characteristics of studies included in the meta-analyses of all-cause standardised mortality

	**Country**	**Study years**	**Study design**	**Migrants (n)**	**Population description**	**Migrant countries or regions of origin**	Quality assessment*(%)
Deckert et al^29^	Germany	1990–2010	Cohort	6378	Ethnic German repatriates from the former Soviet Union	Former Soviet Union	100%
DesMeules et al^20^	Canada	1980–98	Cohort	369 972	Canadian immigrants, including both refugees and non-refugees	Northeast Asia, western Europe, eastern Europe and Russia, south Asia, southeast Asia, the Middle East and Africa, north Africa, the Caribbean, North America, South America, Central America, Oceania, and the Pacific	63%
DesMeules et al^19^	Canada	1980–98	Cohort	369 972	Canadian immigrants, including both refugees and non-refugees	Northeast Asia, western Europe, eastern Europe and Russia, south Asia, southeast Asia, the Middle East and Africa, the Caribbean, North America, South America, Central America, Oceania, and the Pacific	100%
Eschbach et al^22^	USA	1999–2001	Cohort	NR	Foreign-born Hispanics in California and Texas	Mexico, Central America, and South America	88%
Fischbacher et al^23^	Scotland	1997–2003	Cohort	NR	Foreign-born residents of Scotland	England and Wales, Northern Ireland, Ireland, India, Pakistan, Bangladesh, China, Hong Kong, rest of the world	100%
Hammar et al^30^	Sweden	1976–95	Cohort	1994	Finnish migrants to Sweden with ≤20 years residency	Finland	88%
Iwasaki et al^18^	Brazil	1999–2001	Cohort	51 445	First-generation Japanese Brazilians	Japan	100%
Kaucher et al^28^	Germany	1990–2009	Cohort	59 390	Resettlers (ethnic German immigrants) in Germany	Former Soviet Union	88%
Koppenaal et al^4^	Netherlands	1998–99	Cohort	45 889	Asylum seekers to the Netherlands	Multiple	88%
Makarova et al^27^	Germany	2004–10	Cohort	NR	Migrants from the former Soviet Union and Turkey	Former Soviet Union	100%
Ott et al^24^	Germany and Israel	1990–2005	Cohort	34 393 (Germany), 589 388 (Israel)	Regular migrants from the former Soviet Union to Israel and Germany who arrived between 1990 and 2001	Former Soviet Union	88%
Ott et al^25^	Israel	1990–2003	Cohort	926 870	Migrants from the former Soviet Union	Former Soviet Union	88%
Ronellenfitsch et al^21^	Germany	1990–2002	Cohort	34 393	Ethnic German immigrants from the former Soviet Union	Former Soviet Union	100%
van Oostrum et al^26^	Netherlands	2002–05	Cohort	NR	Asylum seekers residing in asylum seeker centres in the Netherlands	West Africa, central Africa, southern Africa, north Africa, east Africa, horn of Africa, central Europe, eastern Europe, southern Europe, the Middle East, southwest Asia, central Asia, east Asia, and south Asia	88%
Verropolou and Tsimbos^17^	Greece	2010–12	Cohort	911 929	International migrants	International migrants from all geographical regions	88%
Wild et al^31^	England and Wales	2001–03	Cohort	NR	Migrants in England and Wales	Ireland, eastern Europe, east Africa, north Africa, west Africa, West Indies, the Middle East, Bangladesh, India, Pakistan, China, and Hong Kong	100%

NR=not reported.

* Quality of included studies was assessed using an adapted version of the Newcastle Ottawa Scale.^16^

**Figure 3:**
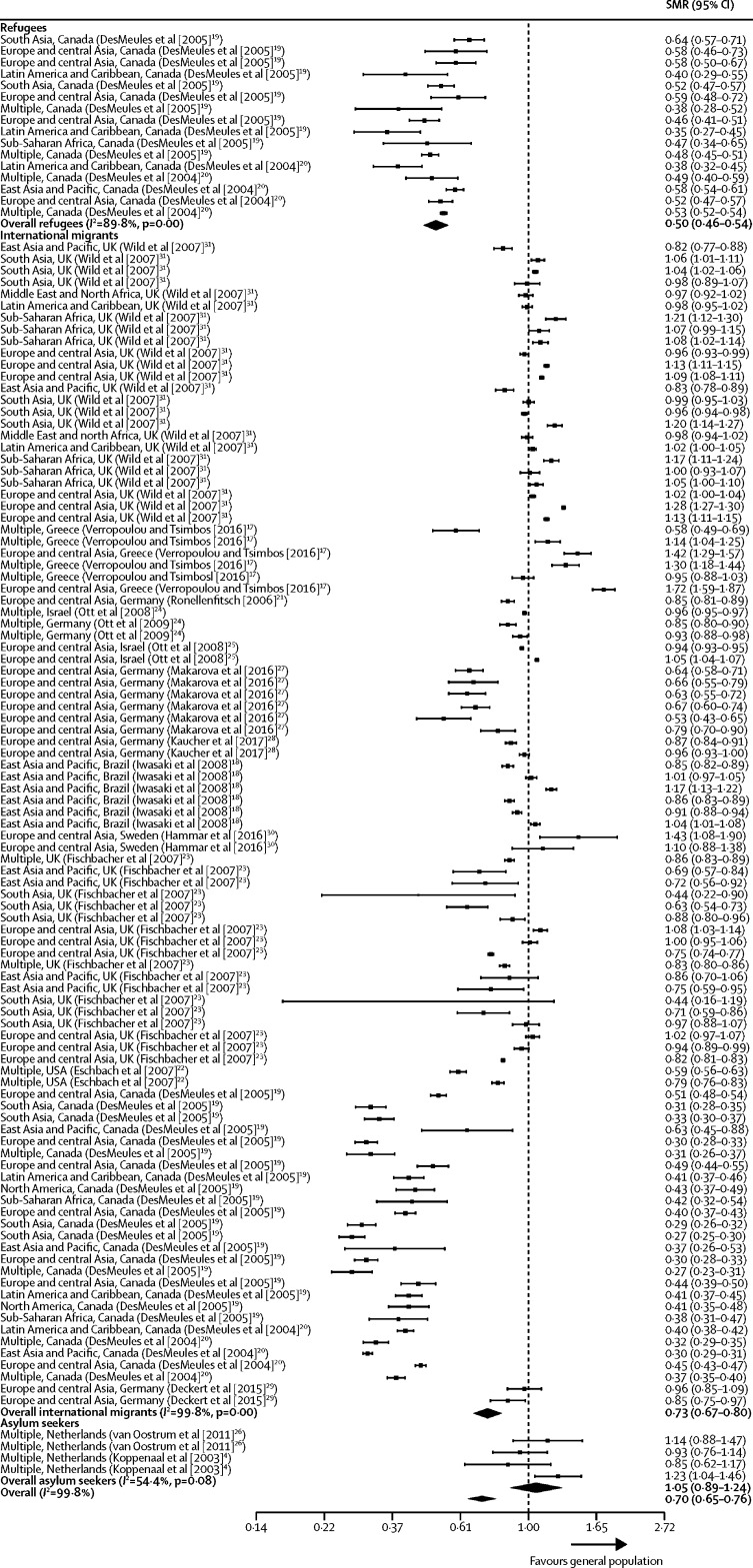
Forest plots of SMRs for all-cause mortality by migrant group Studies are presented as region of origin, country of study (study [year]). 16 studies reported all-cause mortality estimates by migrant group, which included 119 mortality estimates. SMR=standardised mortality ratio.

We assessed all-cause mortality by migrant subgroup, including data on refugees (16 mortality estimates) and asylum seekers (four mortality estimates), and 99 estimates for which migrant subgroup was unspecified. Consistent with the findings for all international migrants, refugees had lower SMRs than the general population (0·50 [0·46–0·54]; *I*^2^=89·8%; figure 3). These all-cause mortality estimates were obtained from two studies^19^, ^20^ done in Canada and included refugees originating from multiple geographical regions of origin. We found no evidence of a mortality advantage for asylum seekers (1·05 [0·89–1·24]; *I*^2^=54·4%), but limited data were available for this group, and only four datapoints from two separate studies done in the Netherlands on migrants from multiple geographical regions of origin.^4^, ^26^

We did a subgroup meta-analysis on all-cause mortality by geographical region of origin and destination (appendix). Summary SMRs for all World Bank regions were less than 1; however, weak statistical evidence indicated a lower mortality in international migrants from sub-Saharan Africa (0·81 [0·59–1·10]; *I*^2^=99·3%) and the Middle East and north Africa (0·98 [0·95–1·01]; *I*^2^=99·3%). We did a post-hoc analysis of all-cause SMRs by World Bank income group in migrant origin countries, investigating a possible association between income at origin and SMRs in the destination country. We only found evidence of reduced mortality in migrants from upper-middle-income countries (0·65 [0·61–0·70]; *I*^2^=0·0%; appendix). Many studies only presented results by geographical region of origin and not country, and therefore this analysis only included 22 datapoints from six high-income countries, five datapoints from two upper middle-income countries, and 12 datapoints from three lower-middle-income countries.

**Figure 4:**
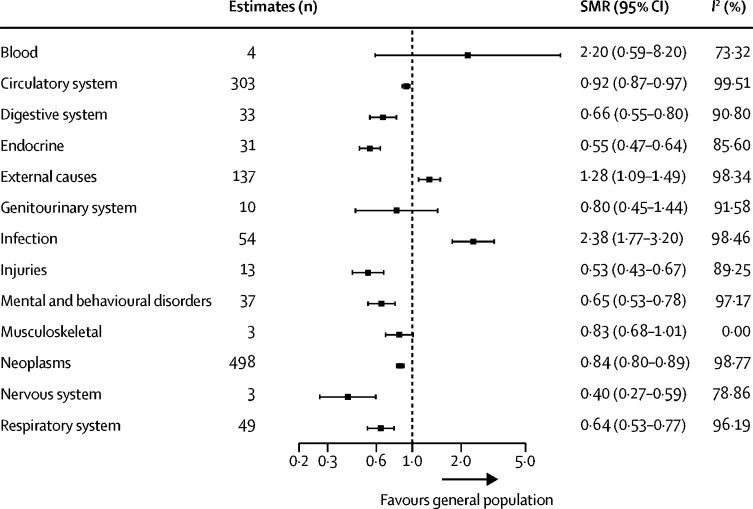
Meta-analysis estimates of SMRs for international migrants by ICD-10 disease category SMR=standardised mortality ratio. ICD-10=International Classification of Diseases, tenth revision.

We included 1175 SMR estimates in our meta-analyses by ICD-10 disease category. Of 13 ICD-10 categories, migrants had a mortality advantage across eight categories (circulatory, digestive, endocrine, injuries, mental and behavioural, neoplasms, nervous, and respiratory) compared with the general population in destination countries (figure 4). No differences in mortality were identified for three disease categories (blood, genitourinary, and musculoskeletal), and migrant mortality was increased for infectious diseases (2·4 [1·8–3·2]; *I*^2^=98·5%) and external causes of mortality (1·3 [1·1–1·5]; *I*^2^=98·3%) compared with the general population.

**Figure 5:**
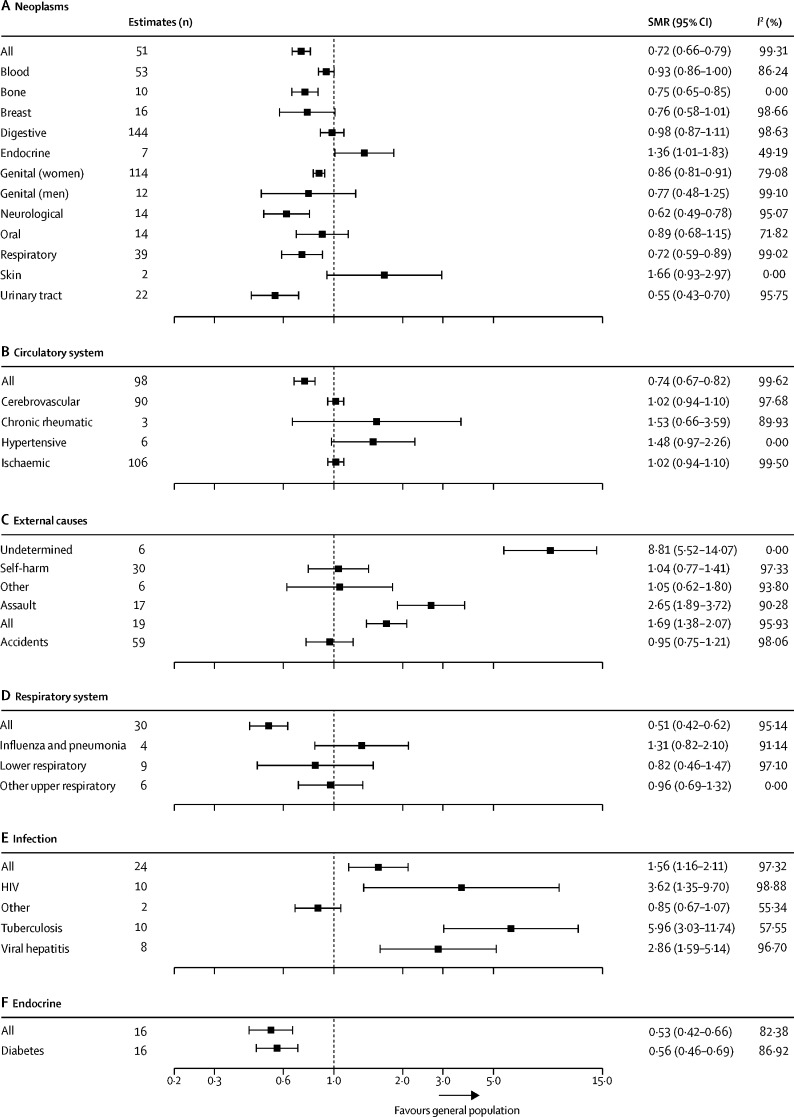
Subgroup analysis of international migrants by ICD-10 subgroup disease category for the six ICD-10 disease categories with the highest total number of SMR estimates Data are presented for neoplasms (A), the circulatory system (B), external causes (C), respiratory diseases (D), infection (E), and endocrine disorders (F). SMR=standardised mortality ratio. ICD-10=International Classification of Diseases, tenth revision.

We did further subgroup analyses, not specified in our protocol, for the six ICD-10 categories with the highest total number of SMR estimates (figure 5). These analyses showed improved or no mortality difference across the majority of subgroups with several important exceptions. Events of undetermined intent were increased in international migrants (8·8 [5·5–14·1]; *I*^2^=0·0%) compared with the general population in host countries, which combined eight point estimates from one study in international migrants in Germany.^29^ There was evidence of increased SMRs for assault (2·7 [1·9–3·7]; *I*^2^=90·3%) among migrants, which combined 17 point estimates from five separate studies done in the USA,^22^ Sweden,^30^ the Netherlands,^4^, ^26^ and Greece.^17^ We also found evidence of increased SMRs for viral hepatitis among migrants (2·9 [1·6–5·1]; *I*^2^=96·7%) from eight estimates in two separate studies in Germany^24^ and Israel,^24^ and the Netherlands.^26^ Mortality for tuberculosis was increased for migrants (6·0 [3·0–11·7]; *I*^2^=57·6%) in three studies in the Netherlands,^26^ Greece,^17^ and Sweden.^32^ Mortality was increased for HIV (3·6 [1·4–9·7]; *I*^2^=98·9%) in four studies in migrants from multiple regions and living in Canada,^19^ the USA,^22^ and the Netherlands.^4^, ^26^

We included 3964 absolute mortality estimates, with 1836 age-standardised, 580 age-adjusted, and 452 age-adjusted and sex-adjusted or standardised estimates. All-cause age-standardised mortality in migrants (of all ages) ranged from 420 per 100 000 population in Nicaraguan migrants living in Costa Rica,^33^ to 874 in male migrants from the former Soviet Union living in Israel.^25^

We did several analyses to examine the balance of the evidence (ie, the proportion of datapoints that represented each migrant type) identified by the review. In a pre-planned analysis, we examined the association between the number of mortality estimates and summary SMR estimates for each ICD-10 category (appendix). Two ICD-10 disease categories had SMR estimates higher than 1, suggesting migrants had higher mortality than the general population: infectious diseases (164 mortality estimates) and external causes of mortality (591). However, the highest total number of estimates were available for neoplasms (n=1643), diseases of the circulatory system (n=969), and external causes of mortality (n=591). Due to the quantity of included data, we also did several post-hoc analyses by geographical region of origin and destination (appendix). Migrants from the Middle East, north Africa, and North America were underrepresented. Migrants to the Middle East and north Africa, sub-Saharan Africa, south Asia, Latin America, and the Caribbean were all underrepresented. The number of mortality estimates was inconsistent across migrant subgroups, with labour migrants and forced migrants (including refugees, asylum seekers, and undocumented migrants) all underrepresented.

High heterogeneity was identified across studies. We investigated this heterogeneity using several pre-specified analyses, including by sex, migrant subgroup, region of origin, and ICD-10 diagnosis and in a post-hoc analysis by ICD-10 subgroup. Although heterogeneity remained high when stratifying by sex or ICD-10 disease category, it was reduced for some analyses when stratified by migrant groups and ICD-10 subgroup.

We examined the quality of included studies using an adapted version of the Newcastle Ottawa Scale, and found that included studies were of high quality with a median score of 89% out of a maximum of 100% (appendix). Only six studies scored less than 50% and therefore we decided not to do our pre-planned quality subgroup meta-analyses. A post-hoc assessment found some evidence for risk of publication bias (appendix) with possible underpublication of results showing lower mortality in migrants.

## Discussion

We found substantial evidence of a mortality advantage in international migrants relative to the general population, with most studies done in high-income countries. This mortality advantage was observed across geographical region of origin and the majority of ICD-10 categories, with the exception of infectious diseases and external causes, in which migrants had increased mortality compared with the general population in destination countries. Infectious disease mortality was increased for viral hepatitis, tuberculosis, and HIV. Assaults and deaths of undetermined intent were increased among migrants for external causes of mortality.

Our results were obtained for international migrants (often reported as foreign-born) in high-income settings and populations reported as foreign born. Data on international migration^34^, ^35^ suggest that of the 258 million international migrants worldwide, 150 million are migrant workers and 5 million are students. Our results are therefore more likely to better reflect outcomes in these groups than other migrant groups. Since the mortality estimates used for our meta-analyses were rarely reported according to migrant subgroup, caution must be taken when generalising these estimates to refugees, asylum seekers, and undocumented migrants, particularly those living in low-income and middle-income countries. These individuals might be at increased risk of mortality, and were underrepresented in research and routine mortality data and thus in our meta-analysis of SMRs. In contrast to many other subgroups of international migrants, a large proportion of refugees move between low-income and middle-income countries, representing an important gap in the existing published literature.

The aggregation of available data on mortality in migrant populations is crucial for comprehensively and rigorously summarising the knowledge base, providing insight with regard to the association between migration and mortality to inform health services, and countering discriminatory or hostile policies.^36^, ^37^ Contrary to the negative representation of migrants in the media as a burden to health systems,^38^ our research provides substantial evidence in support of the mortality advantage of migrants compared with the general population in high-income countries. These results therefore challenge misconceptions and policies that do injustice to migrants, representing them as a risk and burden to health systems and society, and instead highlight positive contributions of migration in these countries.

Previous research^3^, ^39^ has identified several factors that might contribute to improved health outcomes in migrants compared with host populations, and non-migrating peers in countries of origin. Data supporting a healthy migrant hypothesis suggest that healthier migrants might be more likely to choose to migrate, benefit from decisions to migrate, or successfully migrate, and that health is thus a predictor of migration.^40^, ^41^ The mortality advantage of migrants might also be attributed to the so-called salmon bias, whereby migrants return to their countries of origin prior to death.^42^, ^43^ Selective return migration might also occur among migrants who have health or integration challenges, and return to their countries of origin, supporting the so-called unhealthy remigration hypothesis.^39^ However, evidence also suggests that these factors do not entirely explain the mortality advantages, and that other social and cultural mechanisms^44^ are likely to be driving these patterns.^42^ Overestimation of migrant denominator populations could lead to considerable underestimation of mortality rates, particularly in census or registry-based studies that do not remove migrants after they leave a country. A Swedish study^45^ found that accounting for the inflated denominator reduced, but did not eliminate the mortality advantage. Other detailed studies from the UK^46^ and Germany^6^ found that correcting for denominator inflation also did not explain the reduced migrant mortality. A further possible alternative explanation for our findings is residual confounding by age in the analysis of SMRs because the average age of migrants is usually younger than that of host populations. However, we feel that this explanation is unlikely for several reasons. First, the mortality advantage was observed in studies that compared young migrants with young non-migrants in the host country.^22^, ^27^ Second, although the mortality advantage declines with age, it was observed across age categories.^46^, ^47^ Third, using similar methods to this review, we previously found evidence of severely increased SMRs in young marginalised populations including people who have experienced homelessness, substance use, and imprisonment.^15^

However, our study has several limitations. We included studies done in Ethiopia, Guinea, Tanzania, Kenya, Pakistan, and Costa Rica in our review, but Brazil was the only non-high-income country for which migrant data could be included in our meta-analyses of all-cause and cause-specific SMRs. As a result, the mortality advantage reported will be more representative of international migrants in high-income settings, and particularly those studying, working, or who have joined family members. It was not feasible to examine whether the observed mortality advantage changed in relation to time since migration, socioeconomic status, levels of acculturation, over time or before 2001. Despite the quantity of data, we did not attempt to meta-analyse all-cause or cause-specific estimates due to large differences in the reporting of outcomes across studies. Heterogeneity was high across analyses in this study, and our ability to explore this was limited by the scarcity of data on country of origin and migrant subgroup. In particular, all-cause mortality estimates for refugees were provided in only two studies.^19^, ^20^ Previous studies of refugees in humanitarian settings have reported that refugees have excess mortality during and immediately after displacement, but that mortality decreases to levels comparable with the general populations in the months to years afterwards when an effective humanitarian response is in place;^48^ however, we were unable to examine this further in our meta-analyses because data were scarce. Our study also highlights the limited availability of data on mortality in refugees, asylum seekers, and undocumented migrants who originate in and move to low-income and middle-income countries. This is an important limitation of the existing evidence base considering that most of the world's refugees reside in other low-income and middle-income countries close to countries of conflict. We did not include grey literature in our review because the aim was to report on the published evidence base; therefore, data from unpublished registries, local clinics, camps or transit centres are not included, and migrants in transit, thousands of whom die during hazardous journeys, were not included. Our meta-analysis included studies published between January, 2001, and March, 2017. We updated our search from March 31, 2017, to Sept 3, 2018, which yielded ten additional papers^49^, ^50^, ^51^, ^52^, ^53^, ^54^, ^55^, ^56^, ^57^, ^58^ that met our inclusion criteria that had been published since the original search. Of these ten studies, nine were done in high-income countries (two from the same dataset in Belgium), and were focused on international migrants with no disaggregation by subgroup. No studies presented new SMR data eligible for inclusion in our meta-analyses. One study done in Chad^49^ found that absolute mortality in refugees from the Central African Republic was high before and during migration compared with when individuals were settled in refugee camps, but no general comparator population analysis was included.

Our review provides evidence of the mortality advantage of migrants, but this must not be used as a justification for further restricting access to health care for migrant groups, which is an increasing issue in many countries.^36^, ^37^ Health-care needs in migrants vary substantially, as shown by the heterogeneity in our estimates. Morbidity in migrants was not assessed by our study and might be higher in migrant groups, particularly in those who are more marginalised or of lower socioeconomic status. Additionally, we identified areas of higher need including infectious diseases. This is consistent with previous research in low-incidence infectious disease settings in high-income countries.^7^ The increased infectious disease mortality is likely to be a result of incidence in the country of origin and not transmission during or after migration, which has been shown to be low in many high-income settings.^59^, ^60^, ^61^ The increased relative risk of mortality due to infectious disease reflects incidence of these diseases in the countries of origin and when measured in absolute terms is likely to be small. However, the relative increase in death due to HIV, tuberculosis, and viral hepatitis provides evidence for the need to ensure migrants from high-incidence countries have access to high quality treatment and health care, including an affirmative approach to improving linkage to care to reduce risk of death. Further research should examine how health services can reduce mortality and how increasingly restrictive health services in high-income migrant host countries^36^ might contribute to delays in accessing care and completing treatment, leading to poorer and more costly health outcomes.^62^ Therefore, policy makers should aim to develop policies that reflect the varying background nature of this risk in relation to country of origin to ensure appropriate targeting. Summary estimates from our study should be considered in the context of local epidemiology and wherever possible supplemented with local country and migrant group specific estimates to inform local and national screening policies. Infectious disease screening programmes must ensure they are not used to discriminate against, marginalise, or stigmatise migrants, and should only be done when there is evidence of health benefits to migrants.^59^, ^63^

Our finding of raised external causes of mortality is particularly concerning. Evidence of increased relative mortality due to assaults was consistent across several studies in high-income countries, and was driven by mortality estimates for homicide. The implementation of effective interventions in this area will have the benefit of reducing mortality in both migrant and non-migrant populations, and therefore improve the health of the public generally. In addition to these policies, public health efforts to tackle the health threats of racism and xenophobia might reduce assaults on migrants.^64^

The scarcity of analyses by migrant subgroup highlights the need for further research and improved reporting in underrepresented migrant groups. These limitations require more robust and consistent data collection and reporting in migrant health research, as called for by the Global Compact for Safe, Orderly and Regular Migration,^65^ to strengthen the evidence base on migration and health.^66^, ^67^ Data collection on migrants must be supported by a strong information governance, data sharing framework that ensures the appropriate and sensitive use of such data, and prevents its misuse—for example, for immigration enforcement purposes.^68^ Our study also highlights geographical regions with little migration data, particularly for migrants to and from countries in the Middle East and north Africa, representing an important gap in the global literature. Further research should investigate mortality patterns in migrant labour populations in the Middle East and north Africa, for which good records are likely to exist, and for displaced populations as a result of the conflicts in Syria, Iraq, and Yemen. Studies done in low-income and middle-income settings were particularly scarce and concerted efforts to address this deficit are needed.

Our systematic review and meta-analysis is a robust and comprehensive examination of the evidence base on mortality patterns in international migrants. We found that many international migrants in high-income settings had a mortality advantage compared with the general population. We provide further evidence for the positive health benefit that migrants bring to destination countries. Ensuring equitable access to high quality health care globally is crucial, regardless of migrant status, and is essential to facilitate, rather than restrict, entitlement to care for all. Our findings challenge popular misconceptions that migrants in high-income settings are unhealthy and a burden to host societies. Instead, the findings represent an opportunity to reframe the public discourse from one that is dominated by poorly evidenced concerns about the risks associated with migration and health, to one that is evidence-based and overwhelmingly supports the benefits migrants provide to high-income countries.
