# Supplementary material for: Global patterns of mortality in international migrants: a systematic review and meta-analysis
Source: Lancet. 2018 Dec 15;392(10164):2553–66. doi: 10.1016/S0140-6736(18)32781-8 (PMC6294735; doi:10.1016/S0140-6736(18)32781-8)
Supplement: Supplementary appendix [file mmc1.pdf]

THE LANCET

Supplementary appendix

This appendix formed part of the original submission and has been peer reviewed. We post it as supplied by the authors.

Supplement to: Aldridge RW, Nellums LB, Bartlett S, et al. Global patterns of mortality in international migrants: a systematic review and meta-analysis. *Lancet* 2018; published online Dec 4. http://dx.doi.org/10.1016/S0140-6736(18)32781-8.

Supplementary appendix - protocol deviations

We have documented all deviations in our Prospero record so that all deviations are transparently and publicly declared. We were unable to access Global Health database as originally specified and searched Cochrane library and google scholar instead. We performed a subgroup analysis of all cause mortality by world bank income groups that we did not initially specify in our protocol. Because of this additional world bank income subgroup we used World Bank global regions instead of WHO world regions as specified in the protocol. We conducted our meta-analysis in the statistical package R instead of Stata. We removed one secondary outcome from the original protocol - risk of mortality among migrants compared to non-migrants. We removed this secondary outcome because we realised that our primary outcome (Standardised Mortality Ratios -SMR) is a more appropriate than unadjusted risk in terms of accounting for the younger age of migrants compared to host populations - unadjusted risks would inappropriately exaggerate the observed mortality difference towards migrants. We also removed this outcome because after completing our full text review we realised that there was an extensive volume of data on SMRs which reduced need for an additional outcome to increase the meta-analysis sample size. Because of the amount of SMR data by ICD-10 group, we were able to perform an ICD-10 subgroup analysis in order to try and explain heterogeneity within our results that we did not originally specify. Finally, we conducted a post-hoc assessment of the risk of publication bias.

Supplementary appendix - search terms

Medline search terms

1. Human Migration/ or Migration.mp.
2. migrant.mp. or "Transients and Migrants"/
3. Migrant*.mp.
4. "Emigrants and Immigrants"/ or immigra*.mp.
5. Expatriate.mp.
6. Refugees/ or Refugee*.mp.
7. Departee.mp.
8. "Emigration and Immigration"/ or Emigr*.mp.
9. Asylum.mp.
10. Foreign-born.mp.
11. Foreign born.mp.
12. foreign worker.mp.
13. foreign student.mp.
14. International student.mp.
15. human trafficking.mp. or Human Trafficking/
16. people trafficking.mp.

17. trafficking in people.mp.

18. sex trafficking.mp.

19. woman trafficking.mp.

20. child trafficking.mp.

21. trafficked people.mp.

22. trafficked women.mp.

23. trafficked men.mp.

24. trafficked children.mp.

25. 1 or 2 or 3 or 4 or 5 or 6 or 7 or 8 or 9 or 10 or 11 or 12 or 13 or 14 or 15 or 16 or 17 or 18 or 19 or 20 or 21 or 22 or 23 or 24

26. Mortality.mp. or Mortality/

27. standardised mortality ratio.mp. or "Cause of Death"/

28. standardized mortality ratio.mp.

29. SMR.mp.

30. death.mp. or Death/

31. Mortality risk.mp.

32. life table.mp. or Life Tables/

33. fatality.mp.

34. Absolute mortality.mp.

35. Relative mortality.mp.

36. all-cause mortality.mp.

37. ACM.mp.

38. cause specific mortality.mp.

39. CSM.mp.

40. total mortality.mp.

41. 26 or 27 or 28 or 29 or 30 or 31 or 32 or 33 or 34 or 35 or 36 or 37 or 38 or 39 or 40

42. 25 and 41

43. Proteins/ or protein.mp.

44. membrane.mp. or Membranes/

45. cell.mp. or Cells/

46. Animal Migration/ or Birds/ or bird migration.mp.

47. 43 or 44 or 45 or 46

48. 42 not 47

49. limit 48 to yr="2001 -Current"

Supplementary appendix - Figures

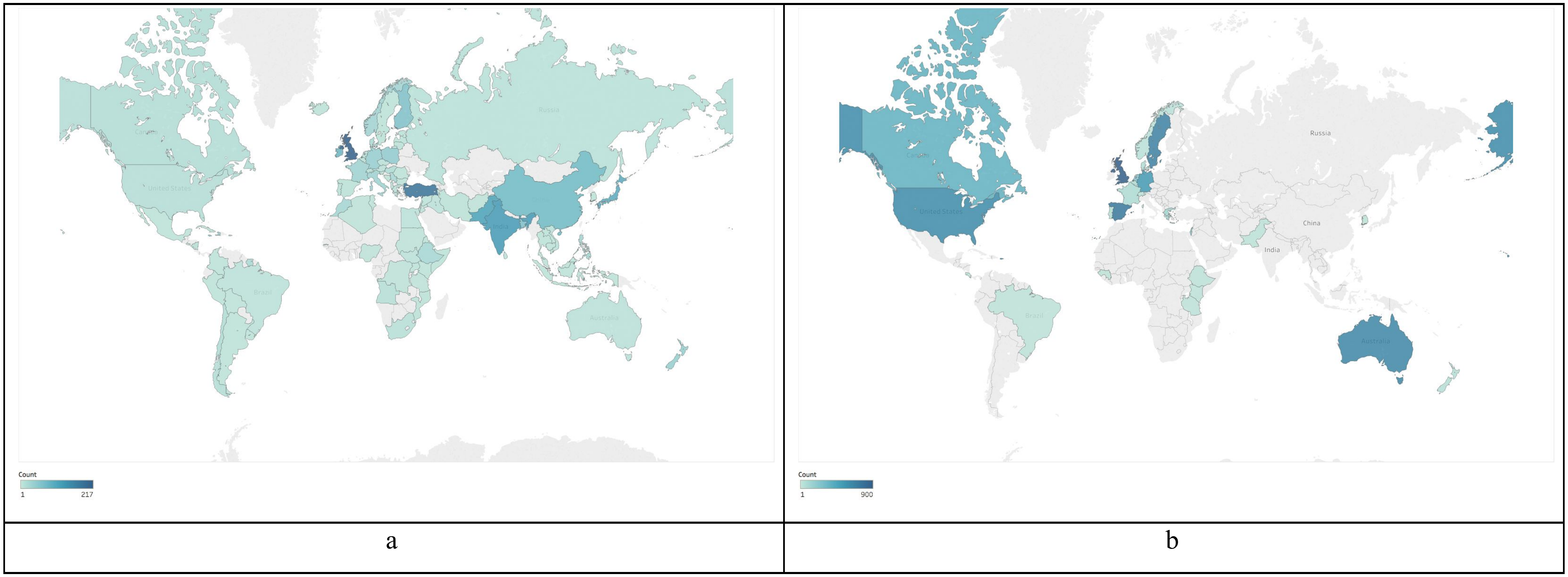

a | b

Figure S1. Countries of origin (a) and destination (b) and international migrant numbers from included studies

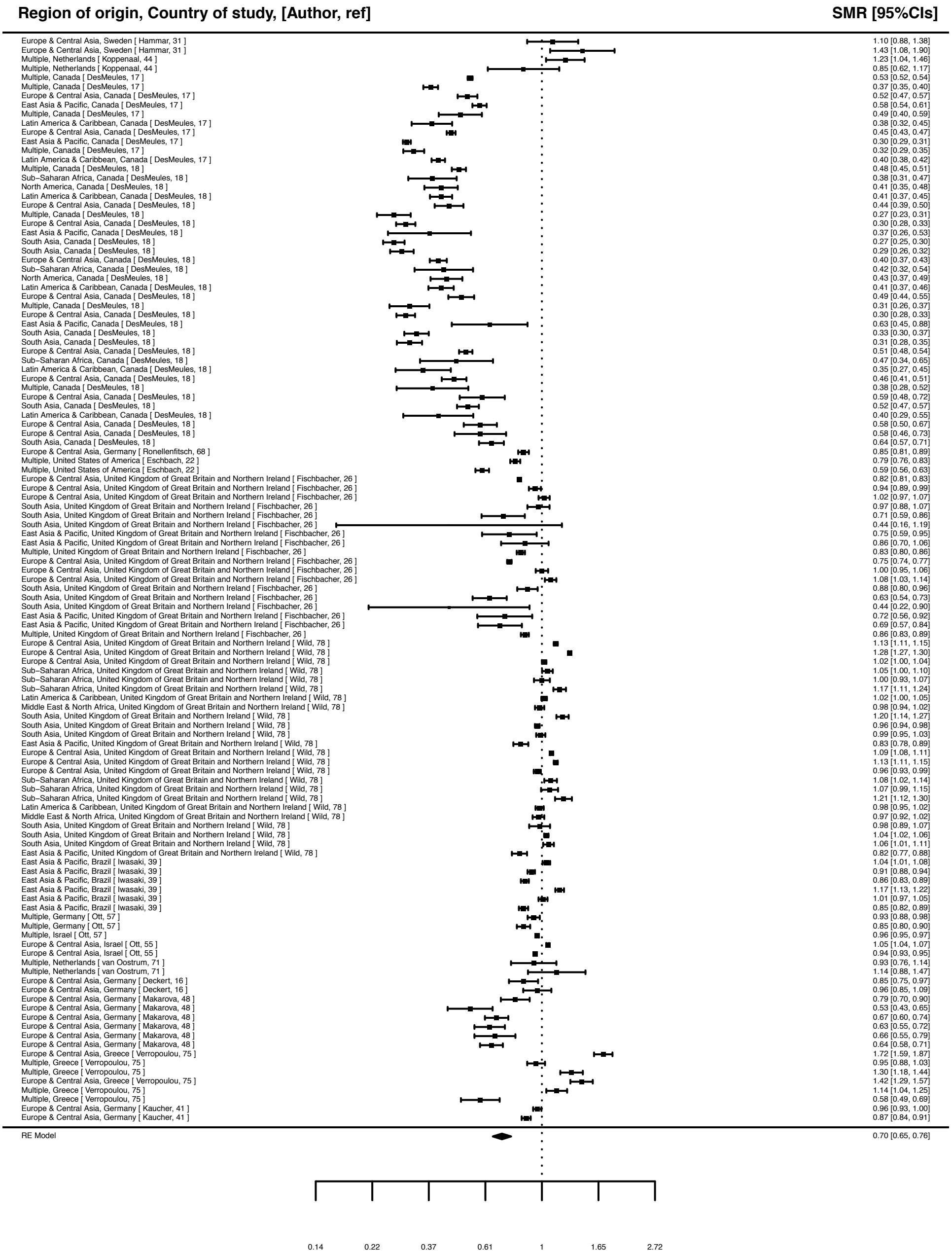
Region of origin, Country of study, [Author, ref]
SMR [95%CIs]
Europe & Central Asia, Sweden [Hammar, 31] 1.10 [0.88, 1.38]
Europe & Central Asia, Sweden [Hammar, 31] 1.43 [1.08, 1.90]
Multiple, Netherlands [Koppenaal, 44] 1.23 [1.04, 1.46]
Multiple, Netherlands [Koppenaal, 44] 0.85 [0.62, 1.17]
Multiple, Canada [DesMeules, 17] 0.53 [0.52, 0.54]
Multiple, Canada [DesMeules, 17] 0.37 [0.35, 0.40]
Europe & Central Asia, Canada [DesMeules, 17] 0.52 [0.47, 0.57]
East Asia & Pacific, Canada [DesMeules, 17] 0.58 [0.54, 0.61]
Multiple, Canada [DesMeules, 17] 0.49 [0.40, 0.59]
Latin America & Caribbean, Canada [DesMeules, 17] 0.38 [0.32, 0.45]
Europe & Central Asia, Canada [DesMeules, 17] 0.45 [0.43, 0.47]
East Asia & Pacific, Canada [DesMeules, 17] 0.30 [0.29, 0.31]
Multiple, Canada [DesMeules, 17] 0.32 [0.29, 0.35]
Latin America & Caribbean, Canada [DesMeules, 17] 0.40 [0.38, 0.42]
Multiple, Canada [DesMeules, 18] 0.48 [0.45, 0.51]
Sub−Saharan Africa, Canada [DesMeules, 18] 0.38 [0.31, 0.47]
North America, Canada [DesMeules, 18] 0.41 [0.35, 0.48]
Latin America & Caribbean, Canada [DesMeules, 18] 0.41 [0.37, 0.45]
Europe & Central Asia, Canada [DesMeules, 18] 0.44 [0.39, 0.50]
Multiple, Canada [DesMeules, 18] 0.27 [0.23, 0.31]
Europe & Central Asia, Canada [DesMeules, 18] 0.30 [0.28, 0.33]
East Asia & Pacific, Canada [DesMeules, 18] 0.37 [0.26, 0.53]
South Asia, Canada [DesMeules, 18] 0.27 [0.25, 0.30]
South Asia, Canada [DesMeules, 18] 0.29 [0.26, 0.32]
Europe & Central Asia, Canada [DesMeules, 18] 0.40 [0.37, 0.43]
Sub−Saharan Africa, Canada [DesMeules, 18] 0.42 [0.32, 0.54]
North America, Canada [DesMeules, 18] 0.43 [0.37, 0.49]
Latin America & Caribbean, Canada [DesMeules, 18] 0.41 [0.37, 0.46]
Europe & Central Asia, Canada [DesMeules, 18] 0.49 [0.44, 0.55]
Multiple, Canada [DesMeules, 18] 0.31 [0.26, 0.37]
Europe & Central Asia, Canada [DesMeules, 18] 0.30 [0.28, 0.33]
East Asia & Pacific, Canada [DesMeules, 18] 0.63 [0.45, 0.88]
South Asia, Canada [DesMeules, 18] 0.33 [0.30, 0.37]
South Asia, Canada [DesMeules, 18] 0.31 [0.28, 0.35]
Europe & Central Asia, Canada [DesMeules, 18] 0.51 [0.48, 0.54]
Sub−Saharan Africa, Canada [DesMeules, 18] 0.47 [0.34, 0.65]
Latin America & Caribbean, Canada [DesMeules, 18] 0.35 [0.27, 0.45]
Europe & Central Asia, Canada [DesMeules, 18] 0.46 [0.41, 0.51]
Multiple, Canada [DesMeules, 18] 0.38 [0.28, 0.52]
Europe & Central Asia, Canada [DesMeules, 18] 0.59 [0.48, 0.72]
South Asia, Canada [DesMeules, 18] 0.52 [0.47, 0.57]
Latin America & Caribbean, Canada [DesMeules, 18] 0.40 [0.29, 0.55]
Europe & Central Asia, Canada [DesMeules, 18] 0.58 [0.50, 0.67]
Europe & Central Asia, Canada [DesMeules, 18] 0.58 [0.46, 0.73]
South Asia, Canada [DesMeules, 18] 0.64 [0.57, 0.71]
Europe & Central Asia, Germany [Ronellenfitsch, 68] 0.85 [0.81, 0.89]
Multiple, United States of America [Eschbach, 22] 0.79 [0.76, 0.83]
Multiple, United States of America [Eschbach, 22] 0.59 [0.56, 0.63]
Europe & Central Asia, United Kingdom of Great Britain and Northern Ireland [Fischbacher, 26] 0.82 [0.81, 0.83]
Europe & Central Asia, United Kingdom of Great Britain and Northern Ireland [Fischbacher, 26] 0.94 [0.89, 0.99]
Europe & Central Asia, United Kingdom of Great Britain and Northern Ireland [Fischbacher, 26] 1.02 [0.97, 1.07]
South Asia, United Kingdom of Great Britain and Northern Ireland [Fischbacher, 26] 0.97 [0.88, 1.07]
South Asia, United Kingdom of Great Britain and Northern Ireland [Fischbacher, 26] 0.71 [0.59, 0.86]
South Asia, United Kingdom of Great Britain and Northern Ireland [Fischbacher, 26] 0.44 [0.16, 1.19]
East Asia & Pacific, United Kingdom of Great Britain and Northern Ireland [Fischbacher, 26] 0.75 [0.59, 0.95]
East Asia & Pacific, United Kingdom of Great Britain and Northern Ireland [Fischbacher, 26] 0.86 [0.70, 1.06]
Multiple, United Kingdom of Great Britain and Northern Ireland [Fischbacher, 26] 0.83 [0.80, 0.86]
Europe & Central Asia, United Kingdom of Great Britain and Northern Ireland [Fischbacher, 26] 0.75 [0.74, 0.77]
Europe & Central Asia, United Kingdom of Great Britain and Northern Ireland [Fischbacher, 26] 1.00 [0.95, 1.06]
Europe & Central Asia, United Kingdom of Great Britain and Northern Ireland [Fischbacher, 26] 1.08 [1.03, 1.14]
South Asia, United Kingdom of Great Britain and Northern Ireland [Fischbacher, 26] 0.88 [0.80, 0.96]
South Asia, United Kingdom of Great Britain and Northern Ireland [Fischbacher, 26] 0.63 [0.54, 0.73]
South Asia, United Kingdom of Great Britain and Northern Ireland [Fischbacher, 26] 0.44 [0.22, 0.90]
East Asia & Pacific, United Kingdom of Great Britain and Northern Ireland [Fischbacher, 26] 0.72 [0.56, 0.92]
East Asia & Pacific, United Kingdom of Great Britain and Northern Ireland [Fischbacher, 26] 0.69 [0.57, 0.84]
Multiple, United Kingdom of Great Britain and Northern Ireland [Fischbacher, 26] 0.86 [0.83, 0.89]
Europe & Central Asia, United Kingdom of Great Britain and Northern Ireland [Wild, 78] 1.13 [1.11, 1.15]
Europe & Central Asia, United Kingdom of Great Britain and Northern Ireland [Wild, 78] 1.28 [1.27, 1.30]
Europe & Central Asia, United Kingdom of Great Britain and Northern Ireland [Wild, 78] 1.02 [1.00, 1.04]
Sub−Saharan Africa, United Kingdom of Great Britain and Northern Ireland [Wild, 78] 1.05 [1.00, 1.10]
Sub−Saharan Africa, United Kingdom of Great Britain and Northern Ireland [Wild, 78] 1.00 [0.93, 1.07]
Sub−Saharan Africa, United Kingdom of Great Britain and Northern Ireland [Wild, 78] 1.17 [1.11, 1.24]
Latin America & Caribbean, United Kingdom of Great Britain and Northern Ireland [Wild, 78] 1.02 [1.00, 1.05]
Middle East & North Africa, United Kingdom of Great Britain and Northern Ireland [Wild, 78] 0.98 [0.94, 1.02]
South Asia, United Kingdom of Great Britain and Northern Ireland [Wild, 78] 1.20 [1.14, 1.27]
South Asia, United Kingdom of Great Britain and Northern Ireland [Wild, 78] 0.96 [0.94, 0.98]
South Asia, United Kingdom of Great Britain and Northern Ireland [Wild, 78] 0.99 [0.95, 1.03]
East Asia & Pacific, United Kingdom of Great Britain and Northern Ireland [Wild, 78] 0.83 [0.78, 0.89]
Europe & Central Asia, United Kingdom of Great Britain and Northern Ireland [Wild, 78] 1.09 [1.08, 1.11]
Europe & Central Asia, United Kingdom of Great Britain and Northern Ireland [Wild, 78] 1.13 [1.11, 1.15]
Europe & Central Asia, United Kingdom of Great Britain and Northern Ireland [Wild, 78] 0.96 [0.93, 0.99]
Sub−Saharan Africa, United Kingdom of Great Britain and Northern Ireland [Wild, 78] 1.08 [1.02, 1.14]
Sub−Saharan Africa, United Kingdom of Great Britain and Northern Ireland [Wild, 78] 1.07 [0.99, 1.15]
Sub−Saharan Africa, United Kingdom of Great Britain and Northern Ireland [Wild, 78] 1.21 [1.12, 1.30]
Latin America & Caribbean, United Kingdom of Great Britain and Northern Ireland [Wild, 78] 0.98 [0.95, 1.02]
Middle East & North Africa, United Kingdom of Great Britain and Northern Ireland [Wild, 78] 0.97 [0.92, 1.02]
South Asia, United Kingdom of Great Britain and Northern Ireland [Wild, 78] 0.98 [0.89, 1.07]
South Asia, United Kingdom of Great Britain and Northern Ireland [Wild, 78] 1.04 [1.02, 1.06]
South Asia, United Kingdom of Great Britain and Northern Ireland [Wild, 78] 1.06 [1.01, 1.11]
East Asia & Pacific, United Kingdom of Great Britain and Northern Ireland [Wild, 78] 0.82 [0.77, 0.88]
East Asia & Pacific, Brazil [Iwasaki, 39] 1.04 [1.01, 1.08]
East Asia & Pacific, Brazil [Iwasaki, 39] 0.91 [0.88, 0.94]
East Asia & Pacific, Brazil [Iwasaki, 39] 0.86 [0.83, 0.89]
East Asia & Pacific, Brazil [Iwasaki, 39] 1.17 [1.13, 1.22]
East Asia & Pacific, Brazil [Iwasaki, 39] 1.01 [0.97, 1.05]
East Asia & Pacific, Brazil [Iwasaki, 39] 0.85 [0.82, 0.89]
Multiple, Germany [Ott, 57] 0.93 [0.88, 0.98]
Multiple, Germany [Ott, 57] 0.85 [0.80, 0.90]
Multiple, Israel [Ott, 57] 0.96 [0.95, 0.97]
Europe & Central Asia, Israel [Ott, 55] 1.05 [1.04, 1.07]
Europe & Central Asia, Israel [Ott, 55] 0.94 [0.93, 0.95]
Multiple, Netherlands [van Oostrum, 71] 0.93 [0.76, 1.14]
Multiple, Netherlands [van Oostrum, 71] 1.14 [0.88, 1.47]
Europe & Central Asia, Germany [Deckert, 16] 0.85 [0.75, 0.97]
Europe & Central Asia, Germany [Deckert, 16] 0.96 [0.85, 1.09]
Europe & Central Asia, Germany [Makarova, 48] 0.79 [0.70, 0.90]
Europe & Central Asia, Germany [Makarova, 48] 0.53 [0.43, 0.65]
Europe & Central Asia, Germany [Makarova, 48] 0.67 [0.60, 0.74]
Europe & Central Asia, Germany [Makarova, 48] 0.63 [0.55, 0.72]
Europe & Central Asia, Germany [Makarova, 48] 0.66 [0.55, 0.79]
Europe & Central Asia, Germany [Makarova, 48] 0.64 [0.58, 0.71]
Europe & Central Asia, Greece [Verropoulou, 75] 1.72 [1.59, 1.87]
Multiple, Greece [Verropoulou, 75] 0.95 [0.88, 1.03]
Multiple, Greece [Verropoulou, 75] 1.30 [1.18, 1.44]
Europe & Central Asia, Greece [Verropoulou, 75] 1.42 [1.29, 1.57]
Multiple, Greece [Verropoulou, 75] 1.14 [1.04, 1.25]
Multiple, Greece [Verropoulou, 75] 0.58 [0.49, 0.69]
Europe & Central Asia, Germany [Kaucher, 41] 0.96 [0.93, 1.00]
Europe & Central Asia, Germany [Kaucher, 41] 0.87 [0.84, 0.91]
RE Model 0.70 [0.65, 0.76]
0.14
0.22
0.37
0.61
1
1.65
2.72

Figure S2. Forest plots of SMRs for all-cause mortality both sexes combined

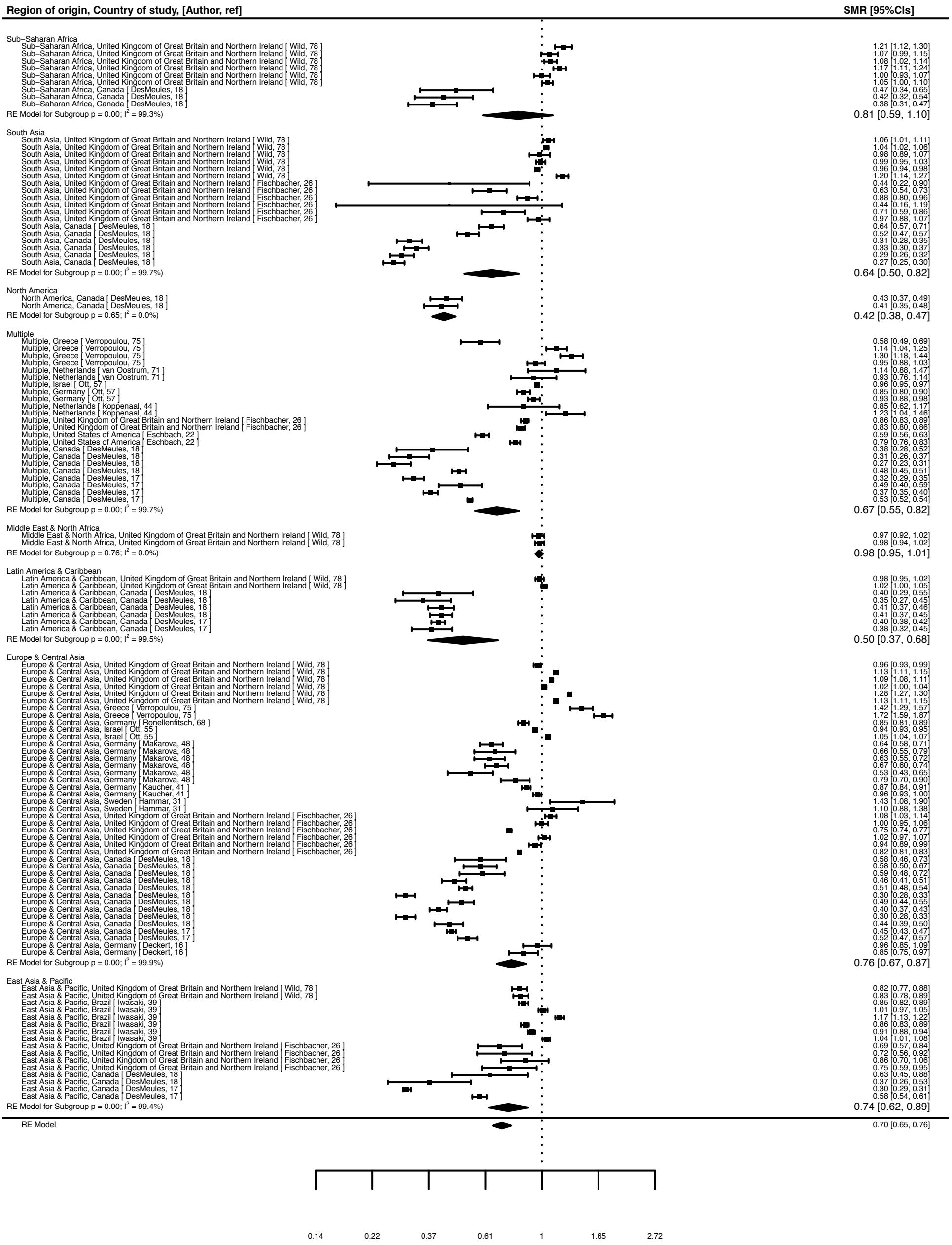
Region of origin, Country of study, [Author, ref]
SMR [95%CIs]
Sub−Saharan Africa
Sub−Saharan Africa, United Kingdom of Great Britain and Northern Ireland [Wild, 78] 1.21 [1.12, 1.30]
Sub−Saharan Africa, United Kingdom of Great Britain and Northern Ireland [Wild, 78] 1.07 [0.99, 1.15]
Sub−Saharan Africa, United Kingdom of Great Britain and Northern Ireland [Wild, 78] 1.08 [1.02, 1.14]
Sub−Saharan Africa, United Kingdom of Great Britain and Northern Ireland [Wild, 78] 1.17 [1.11, 1.24]
Sub−Saharan Africa, United Kingdom of Great Britain and Northern Ireland [Wild, 78] 1.00 [0.93, 1.07]
Sub−Saharan Africa, United Kingdom of Great Britain and Northern Ireland [Wild, 78] 1.05 [1.00, 1.10]
Sub−Saharan Africa, Canada [DesMeules, 18] 0.47 [0.34, 0.65]
Sub−Saharan Africa, Canada [DesMeules, 18] 0.42 [0.32, 0.54]
Sub−Saharan Africa, Canada [DesMeules, 18] 0.38 [0.31, 0.47]
RE Model for Subgroup p = 0.00; I^2 = 99.3%) 0.81 [0.59, 1.10]
South Asia
South Asia, United Kingdom of Great Britain and Northern Ireland [Wild, 78] 1.06 [1.01, 1.11]
South Asia, United Kingdom of Great Britain and Northern Ireland [Wild, 78] 1.04 [1.02, 1.06]
South Asia, United Kingdom of Great Britain and Northern Ireland [Wild, 78] 0.98 [0.89, 1.07]
South Asia, United Kingdom of Great Britain and Northern Ireland [Wild, 78] 0.99 [0.95, 1.03]
South Asia, United Kingdom of Great Britain and Northern Ireland [Wild, 78] 0.96 [0.94, 0.98]
South Asia, United Kingdom of Great Britain and Northern Ireland [Wild, 78] 1.20 [1.14, 1.27]
South Asia, United Kingdom of Great Britain and Northern Ireland [Fischbacher, 26] 0.44 [0.22, 0.90]
South Asia, United Kingdom of Great Britain and Northern Ireland [Fischbacher, 26] 0.63 [0.54, 0.73]
South Asia, United Kingdom of Great Britain and Northern Ireland [Fischbacher, 26] 0.88 [0.80, 0.96]
South Asia, United Kingdom of Great Britain and Northern Ireland [Fischbacher, 26] 0.44 [0.16, 1.19]
South Asia, United Kingdom of Great Britain and Northern Ireland [Fischbacher, 26] 0.71 [0.59, 0.86]
South Asia, United Kingdom of Great Britain and Northern Ireland [Fischbacher, 26] 0.97 [0.88, 1.07]
South Asia, Canada [DesMeules, 18] 0.64 [0.57, 0.71]
South Asia, Canada [DesMeules, 18] 0.52 [0.47, 0.57]
South Asia, Canada [DesMeules, 18] 0.31 [0.28, 0.35]
South Asia, Canada [DesMeules, 18] 0.33 [0.30, 0.37]
South Asia, Canada [DesMeules, 18] 0.29 [0.26, 0.32]
South Asia, Canada [DesMeules, 18] 0.27 [0.25, 0.30]
RE Model for Subgroup p = 0.00; I^2 = 99.7%) 0.64 [0.50, 0.82]
North America
North America, Canada [DesMeules, 18] 0.43 [0.37, 0.49]
North America, Canada [DesMeules, 18] 0.41 [0.35, 0.48]
RE Model for Subgroup p = 0.65; I^2 = 0.0%) 0.42 [0.38, 0.47]
Multiple
Multiple, Greece [Verropoulou, 75] 0.58 [0.49, 0.69]
Multiple, Greece [Verropoulou, 75] 1.14 [1.04, 1.25]
Multiple, Greece [Verropoulou, 75] 1.30 [1.18, 1.44]
Multiple, Greece [Verropoulou, 75] 0.95 [0.88, 1.03]
Multiple, Netherlands [van Oostrum, 71] 1.14 [0.88, 1.47]
Multiple, Netherlands [van Oostrum, 71] 0.93 [0.76, 1.14]
Multiple, Israel [Ott, 57] 0.96 [0.95, 0.97]
Multiple, Germany [Ott, 57] 0.85 [0.80, 0.90]
Multiple, Germany [Ott, 57] 0.93 [0.88, 0.98]
Multiple, Netherlands [Koppenaal, 44] 0.85 [0.62, 1.17]
Multiple, Netherlands [Koppenaal, 44] 1.23 [1.04, 1.46]
Multiple, United Kingdom of Great Britain and Northern Ireland [Fischbacher, 26] 0.86 [0.83, 0.89]
Multiple, United Kingdom of Great Britain and Northern Ireland [Fischbacher, 26] 0.83 [0.80, 0.86]
Multiple, United States of America [Eschbach, 22] 0.59 [0.56, 0.63]
Multiple, United States of America [Eschbach, 22] 0.79 [0.76, 0.83]
Multiple, Canada [DesMeules, 18] 0.38 [0.28, 0.52]
Multiple, Canada [DesMeules, 18] 0.31 [0.26, 0.37]
Multiple, Canada [DesMeules, 18] 0.27 [0.23, 0.31]
Multiple, Canada [DesMeules, 18] 0.48 [0.45, 0.51]
Multiple, Canada [DesMeules, 17] 0.32 [0.29, 0.35]
Multiple, Canada [DesMeules, 17] 0.49 [0.40, 0.59]
Multiple, Canada [DesMeules, 17] 0.37 [0.35, 0.40]
Multiple, Canada [DesMeules, 17] 0.53 [0.52, 0.54]
RE Model for Subgroup p = 0.00; I^2 = 99.7%) 0.67 [0.55, 0.82]
Middle East & North Africa
Middle East & North Africa, United Kingdom of Great Britain and Northern Ireland [Wild, 78] 0.97 [0.92, 1.02]
Middle East & North Africa, United Kingdom of Great Britain and Northern Ireland [Wild, 78] 0.98 [0.94, 1.02]
RE Model for Subgroup p = 0.76; I^2 = 0.0%) 0.98 [0.95, 1.01]
Latin America & Caribbean
Latin America & Caribbean, United Kingdom of Great Britain and Northern Ireland [Wild, 78] 0.98 [0.95, 1.02]
Latin America & Caribbean, United Kingdom of Great Britain and Northern Ireland [Wild, 78] 1.02 [1.00, 1.05]
Latin America & Caribbean, Canada [DesMeules, 18] 0.40 [0.29, 0.55]
Latin America & Caribbean, Canada [DesMeules, 18] 0.35 [0.27, 0.45]
Latin America & Caribbean, Canada [DesMeules, 18] 0.41 [0.37, 0.46]
Latin America & Caribbean, Canada [DesMeules, 18] 0.41 [0.37, 0.45]
Latin America & Caribbean, Canada [DesMeules, 17] 0.40 [0.38, 0.42]
Latin America & Caribbean, Canada [DesMeules, 17] 0.38 [0.32, 0.45]
RE Model for Subgroup p = 0.00; I^2 = 99.5%) 0.50 [0.37, 0.68]
Europe & Central Asia
Europe & Central Asia, United Kingdom of Great Britain and Northern Ireland [Wild, 78] 0.96 [0.93, 0.99]
Europe & Central Asia, United Kingdom of Great Britain and Northern Ireland [Wild, 78] 1.13 [1.11, 1.15]
Europe & Central Asia, United Kingdom of Great Britain and Northern Ireland [Wild, 78] 1.09 [1.08, 1.11]
Europe & Central Asia, United Kingdom of Great Britain and Northern Ireland [Wild, 78] 1.02 [1.00, 1.04]
Europe & Central Asia, United Kingdom of Great Britain and Northern Ireland [Wild, 78] 1.28 [1.27, 1.30]
Europe & Central Asia, United Kingdom of Great Britain and Northern Ireland [Wild, 78] 1.13 [1.11, 1.15]
Europe & Central Asia, Greece [Verropoulou, 75] 1.42 [1.29, 1.57]
Europe & Central Asia, Greece [Verropoulou, 75] 1.72 [1.59, 1.87]
Europe & Central Asia, Germany [Ronellenfitsch, 68] 0.85 [0.81, 0.89]
Europe & Central Asia, Israel [Ott, 55] 0.94 [0.93, 0.95]
Europe & Central Asia, Israel [Ott, 55] 1.05 [1.04, 1.07]
Europe & Central Asia, Germany [Makarova, 48] 0.64 [0.58, 0.71]
Europe & Central Asia, Germany [Makarova, 48] 0.66 [0.55, 0.79]
Europe & Central Asia, Germany [Makarova, 48] 0.63 [0.55, 0.72]
Europe & Central Asia, Germany [Makarova, 48] 0.67 [0.60, 0.74]
Europe & Central Asia, Germany [Makarova, 48] 0.53 [0.43, 0.65]
Europe & Central Asia, Germany [Makarova, 48] 0.79 [0.70, 0.90]
Europe & Central Asia, Germany [Kaucher, 41] 0.87 [0.84, 0.91]
Europe & Central Asia, Germany [Kaucher, 41] 0.96 [0.93, 1.00]
Europe & Central Asia, Sweden [Hammar, 31] 1.43 [1.08, 1.90]
Europe & Central Asia, Sweden [Hammar, 31] 1.10 [0.88, 1.38]
Europe & Central Asia, United Kingdom of Great Britain and Northern Ireland [Fischbacher, 26] 1.08 [1.03, 1.14]
Europe & Central Asia, United Kingdom of Great Britain and Northern Ireland [Fischbacher, 26] 1.00 [0.95, 1.06]
Europe & Central Asia, United Kingdom of Great Britain and Northern Ireland [Fischbacher, 26] 0.75 [0.74, 0.77]
Europe & Central Asia, United Kingdom of Great Britain and Northern Ireland [Fischbacher, 26] 1.02 [0.97, 1.07]
Europe & Central Asia, United Kingdom of Great Britain and Northern Ireland [Fischbacher, 26] 0.94 [0.89, 0.99]
Europe & Central Asia, United Kingdom of Great Britain and Northern Ireland [Fischbacher, 26] 0.82 [0.81, 0.83]
Europe & Central Asia, Canada [DesMeules, 18] 0.58 [0.46, 0.73]
Europe & Central Asia, Canada [DesMeules, 18] 0.58 [0.50, 0.67]
Europe & Central Asia, Canada [DesMeules, 18] 0.59 [0.48, 0.72]
Europe & Central Asia, Canada [DesMeules, 18] 0.46 [0.41, 0.51]
Europe & Central Asia, Canada [DesMeules, 18] 0.51 [0.48, 0.54]
Europe & Central Asia, Canada [DesMeules, 18] 0.30 [0.28, 0.33]
Europe & Central Asia, Canada [DesMeules, 18] 0.49 [0.44, 0.55]
Europe & Central Asia, Canada [DesMeules, 18] 0.40 [0.37, 0.43]
Europe & Central Asia, Canada [DesMeules, 18] 0.30 [0.28, 0.33]
Europe & Central Asia, Canada [DesMeules, 18] 0.44 [0.39, 0.50]
Europe & Central Asia, Canada [DesMeules, 17] 0.45 [0.43, 0.47]
Europe & Central Asia, Canada [DesMeules, 17] 0.52 [0.47, 0.57]
Europe & Central Asia, Germany [Deckert, 16] 0.96 [0.85, 1.09]
Europe & Central Asia, Germany [Deckert, 16] 0.85 [0.75, 0.97]
RE Model for Subgroup p = 0.00; I^2 = 99.9%) 0.76 [0.67, 0.87]
East Asia & Pacific
East Asia & Pacific, United Kingdom of Great Britain and Northern Ireland [Wild, 78] 0.82 [0.77, 0.88]
East Asia & Pacific, United Kingdom of Great Britain and Northern Ireland [Wild, 78] 0.83 [0.78, 0.89]
East Asia & Pacific, Brazil [Iwasaki, 39] 0.85 [0.82, 0.89]
East Asia & Pacific, Brazil [Iwasaki, 39] 1.01 [0.97, 1.05]
East Asia & Pacific, Brazil [Iwasaki, 39] 1.17 [1.13, 1.22]
East Asia & Pacific, Brazil [Iwasaki, 39] 0.86 [0.83, 0.89]
East Asia & Pacific, Brazil [Iwasaki, 39] 0.91 [0.88, 0.94]
East Asia & Pacific, Brazil [Iwasaki, 39] 1.04 [1.01, 1.08]
East Asia & Pacific, United Kingdom of Great Britain and Northern Ireland [Fischbacher, 26] 0.69 [0.57, 0.84]
East Asia & Pacific, United Kingdom of Great Britain and Northern Ireland [Fischbacher, 26] 0.72 [0.56, 0.92]
East Asia & Pacific, United Kingdom of Great Britain and Northern Ireland [Fischbacher, 26] 0.86 [0.70, 1.06]
East Asia & Pacific, United Kingdom of Great Britain and Northern Ireland [Fischbacher, 26] 0.75 [0.59, 0.95]
East Asia & Pacific, Canada [DesMeules, 18] 0.63 [0.45, 0.88]
East Asia & Pacific, Canada [DesMeules, 18] 0.37 [0.26, 0.53]
East Asia & Pacific, Canada [DesMeules, 17] 0.30 [0.29, 0.31]
East Asia & Pacific, Canada [DesMeules, 17] 0.58 [0.54, 0.61]
RE Model for Subgroup p = 0.00; I^2 = 99.4%) 0.74 [0.62, 0.89]
RE Model 0.70 [0.65, 0.76]
0.14
0.22
0.37
0.61
1
1.65
2.72

Figure S3. Forest plots of SMRs for all-cause mortality by region of origin

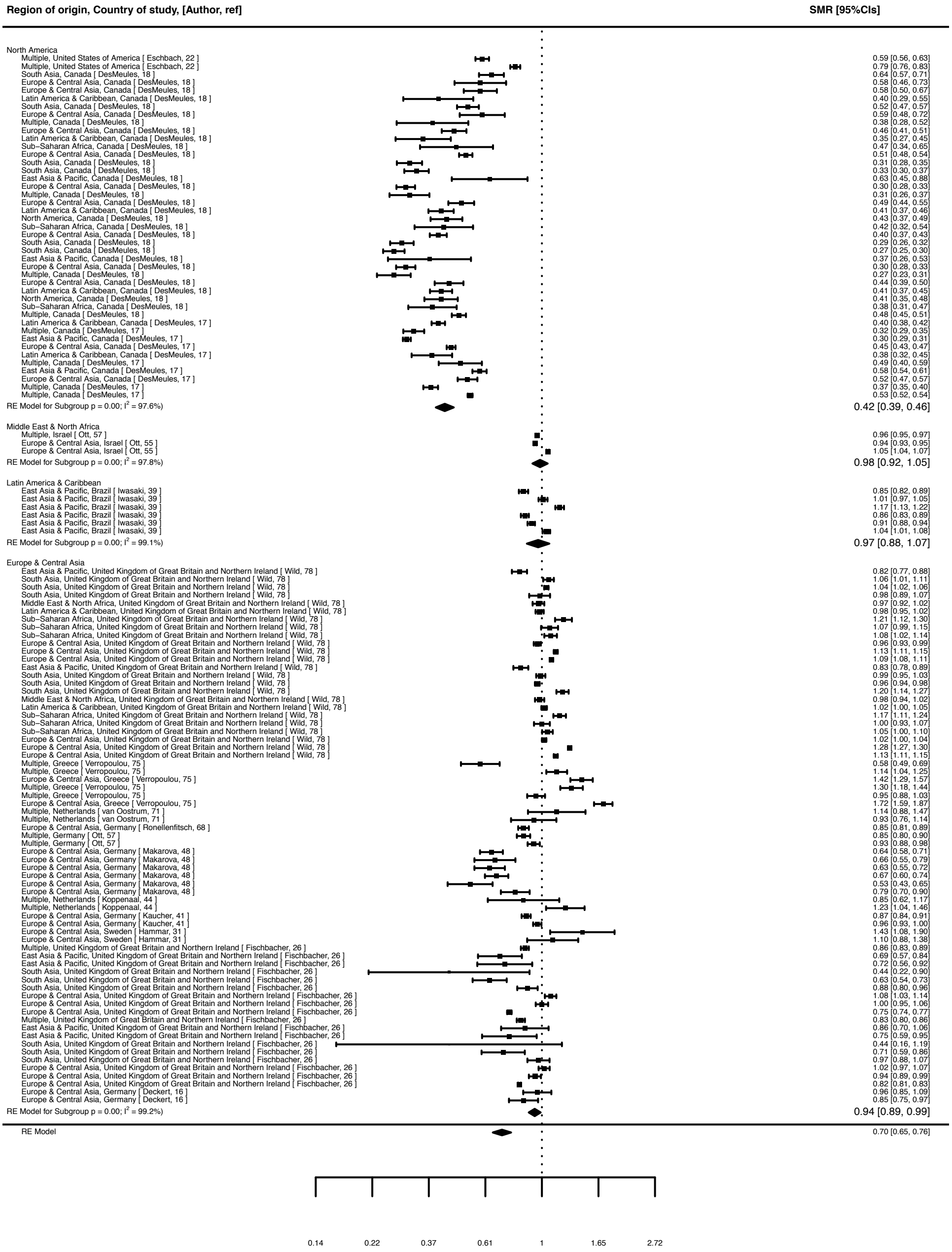

Region of origin, Country of study, [Author, ref]
SMR [95%CIs]
North America
Multiple, United States of America [Eschbach, 22] 0.59 [0.56, 0.63]
Multiple, United States of America [Eschbach, 22] 0.79 [0.76, 0.83]
South Asia, Canada [DesMeules, 18] 0.64 [0.57, 0.71]
Europe & Central Asia, Canada [DesMeules, 18] 0.58 [0.46, 0.73]
Europe & Central Asia, Canada [DesMeules, 18] 0.58 [0.50, 0.67]
Latin America & Caribbean, Canada [DesMeules, 18] 0.40 [0.29, 0.55]
South Asia, Canada [DesMeules, 18] 0.52 [0.47, 0.57]
Europe & Central Asia, Canada [DesMeules, 18] 0.59 [0.48, 0.72]
Multiple, Canada [DesMeules, 18] 0.38 [0.28, 0.52]
Europe & Central Asia, Canada [DesMeules, 18] 0.46 [0.41, 0.51]
Latin America & Caribbean, Canada [DesMeules, 18] 0.35 [0.27, 0.45]
Sub−Saharan Africa, Canada [DesMeules, 18] 0.47 [0.34, 0.65]
Europe & Central Asia, Canada [DesMeules, 18] 0.51 [0.48, 0.54]
South Asia, Canada [DesMeules, 18] 0.31 [0.28, 0.35]
South Asia, Canada [DesMeules, 18] 0.33 [0.30, 0.37]
East Asia & Pacific, Canada [DesMeules, 18] 0.63 [0.45, 0.88]
Europe & Central Asia, Canada [DesMeules, 18] 0.30 [0.28, 0.33]
Multiple, Canada [DesMeules, 18] 0.31 [0.26, 0.37]
Europe & Central Asia, Canada [DesMeules, 18] 0.49 [0.44, 0.55]
Latin America & Caribbean, Canada [DesMeules, 18] 0.41 [0.37, 0.46]
North America, Canada [DesMeules, 18] 0.43 [0.37, 0.49]
Sub−Saharan Africa, Canada [DesMeules, 18] 0.42 [0.32, 0.54]
Europe & Central Asia, Canada [DesMeules, 18] 0.40 [0.37, 0.43]
South Asia, Canada [DesMeules, 18] 0.29 [0.26, 0.32]
South Asia, Canada [DesMeules, 18] 0.27 [0.25, 0.30]
East Asia & Pacific, Canada [DesMeules, 18] 0.37 [0.26, 0.53]
Europe & Central Asia, Canada [DesMeules, 18] 0.30 [0.28, 0.33]
Multiple, Canada [DesMeules, 18] 0.27 [0.23, 0.31]
Europe & Central Asia, Canada [DesMeules, 18] 0.44 [0.39, 0.50]
Latin America & Caribbean, Canada [DesMeules, 18] 0.41 [0.37, 0.45]
North America, Canada [DesMeules, 18] 0.41 [0.35, 0.48]
Sub−Saharan Africa, Canada [DesMeules, 18] 0.38 [0.31, 0.47]
Multiple, Canada [DesMeules, 18] 0.48 [0.45, 0.51]
Latin America & Caribbean, Canada [DesMeules, 17] 0.40 [0.38, 0.42]
Multiple, Canada [DesMeules, 17] 0.32 [0.29, 0.35]
East Asia & Pacific, Canada [DesMeules, 17] 0.30 [0.29, 0.31]
Europe & Central Asia, Canada [DesMeules, 17] 0.45 [0.43, 0.47]
Latin America & Caribbean, Canada [DesMeules, 17] 0.38 [0.32, 0.45]
Multiple, Canada [DesMeules, 17] 0.49 [0.40, 0.59]
East Asia & Pacific, Canada [DesMeules, 17] 0.58 [0.54, 0.61]
Europe & Central Asia, Canada [DesMeules, 17] 0.52 [0.47, 0.57]
Multiple, Canada [DesMeules, 17] 0.37 [0.35, 0.40]
Multiple, Canada [DesMeules, 17] 0.53 [0.52, 0.54]
RE Model for Subgroup p = 0.00; I2 = 97.6%) 0.42 [0.39, 0.46]
Middle East & North Africa
Multiple, Israel [Ott, 57] 0.96 [0.95, 0.97]
Europe & Central Asia, Israel [Ott, 55] 0.94 [0.93, 0.95]
Europe & Central Asia, Israel [Ott, 55] 1.05 [1.04, 1.07]
RE Model for Subgroup p = 0.00; I2 = 97.8%) 0.98 [0.92, 1.05]
Latin America & Caribbean
East Asia & Pacific, Brazil [Iwasaki, 39] 0.85 [0.82, 0.89]
East Asia & Pacific, Brazil [Iwasaki, 39] 1.01 [0.97, 1.05]
East Asia & Pacific, Brazil [Iwasaki, 39] 1.17 [1.13, 1.22]
East Asia & Pacific, Brazil [Iwasaki, 39] 0.86 [0.83, 0.89]
East Asia & Pacific, Brazil [Iwasaki, 39] 0.91 [0.88, 0.94]
East Asia & Pacific, Brazil [Iwasaki, 39] 1.04 [1.01, 1.08]
RE Model for Subgroup p = 0.00; I2 = 99.1%) 0.97 [0.88, 1.07]
Europe & Central Asia
East Asia & Pacific, United Kingdom of Great Britain and Northern Ireland [Wild, 78] 0.82 [0.77, 0.88]
South Asia, United Kingdom of Great Britain and Northern Ireland [Wild, 78] 1.06 [1.01, 1.11]
South Asia, United Kingdom of Great Britain and Northern Ireland [Wild, 78] 1.04 [1.02, 1.06]
South Asia, United Kingdom of Great Britain and Northern Ireland [Wild, 78] 0.98 [0.89, 1.07]
Middle East & North Africa, United Kingdom of Great Britain and Northern Ireland [Wild, 78] 0.97 [0.92, 1.02]
Latin America & Caribbean, United Kingdom of Great Britain and Northern Ireland [Wild, 78] 0.98 [0.95, 1.02]
Sub−Saharan Africa, United Kingdom of Great Britain and Northern Ireland [Wild, 78] 1.21 [1.12, 1.30]
Sub−Saharan Africa, United Kingdom of Great Britain and Northern Ireland [Wild, 78] 1.07 [0.99, 1.15]
Sub−Saharan Africa, United Kingdom of Great Britain and Northern Ireland [Wild, 78] 1.08 [1.02, 1.14]
Europe & Central Asia, United Kingdom of Great Britain and Northern Ireland [Wild, 78] 0.96 [0.93, 0.99]
Europe & Central Asia, United Kingdom of Great Britain and Northern Ireland [Wild, 78] 1.13 [1.11, 1.15]
Europe & Central Asia, United Kingdom of Great Britain and Northern Ireland [Wild, 78] 1.09 [1.08, 1.11]
East Asia & Pacific, United Kingdom of Great Britain and Northern Ireland [Wild, 78] 0.83 [0.78, 0.89]
South Asia, United Kingdom of Great Britain and Northern Ireland [Wild, 78] 0.99 [0.95, 1.03]
South Asia, United Kingdom of Great Britain and Northern Ireland [Wild, 78] 0.96 [0.94, 0.98]
South Asia, United Kingdom of Great Britain and Northern Ireland [Wild, 78] 1.20 [1.14, 1.27]
Middle East & North Africa, United Kingdom of Great Britain and Northern Ireland [Wild, 78] 0.98 [0.94, 1.02]
Latin America & Caribbean, United Kingdom of Great Britain and Northern Ireland [Wild, 78] 1.02 [1.00, 1.05]
Sub−Saharan Africa, United Kingdom of Great Britain and Northern Ireland [Wild, 78] 1.17 [1.11, 1.24]
Sub−Saharan Africa, United Kingdom of Great Britain and Northern Ireland [Wild, 78] 1.00 [0.93, 1.07]
Sub−Saharan Africa, United Kingdom of Great Britain and Northern Ireland [Wild, 78] 1.05 [1.00, 1.10]
Europe & Central Asia, United Kingdom of Great Britain and Northern Ireland [Wild, 78] 1.02 [1.00, 1.04]
Europe & Central Asia, United Kingdom of Great Britain and Northern Ireland [Wild, 78] 1.28 [1.27, 1.30]
Europe & Central Asia, United Kingdom of Great Britain and Northern Ireland [Wild, 78] 1.13 [1.11, 1.15]
Multiple, Greece [Verropoulou, 75] 0.58 [0.49, 0.69]
Multiple, Greece [Verropoulou, 75] 1.14 [1.04, 1.25]
Europe & Central Asia, Greece [Verropoulou, 75] 1.42 [1.29, 1.57]
Multiple, Greece [Verropoulou, 75] 1.30 [1.18, 1.44]
Multiple, Greece [Verropoulou, 75] 0.95 [0.88, 1.03]
Europe & Central Asia, Greece [Verropoulou, 75] 1.72 [1.59, 1.87]
Multiple, Netherlands [van Oostrum, 71] 1.14 [0.88, 1.47]
Multiple, Netherlands [van Oostrum, 71] 0.93 [0.76, 1.14]
Europe & Central Asia, Germany [Ronellenfitsch, 68] 0.85 [0.81, 0.89]
Multiple, Germany [Ott, 57] 0.85 [0.80, 0.90]
Multiple, Germany [Ott, 57] 0.93 [0.88, 0.98]
Europe & Central Asia, Germany [Makarova, 48] 0.64 [0.58, 0.71]
Europe & Central Asia, Germany [Makarova, 48] 0.66 [0.55, 0.79]
Europe & Central Asia, Germany [Makarova, 48] 0.63 [0.55, 0.72]
Europe & Central Asia, Germany [Makarova, 48] 0.67 [0.60, 0.74]
Europe & Central Asia, Germany [Makarova, 48] 0.53 [0.43, 0.65]
Europe & Central Asia, Germany [Makarova, 48] 0.79 [0.70, 0.90]
Multiple, Netherlands [Koppenaal, 44] 0.85 [0.62, 1.17]
Multiple, Netherlands [Koppenaal, 44] 1.23 [1.04, 1.46]
Europe & Central Asia, Germany [Kaucher, 41] 0.87 [0.84, 0.91]
Europe & Central Asia, Germany [Kaucher, 41] 0.96 [0.93, 1.00]
Europe & Central Asia, Sweden [Hammar, 31] 1.43 [1.08, 1.90]
Europe & Central Asia, Sweden [Hammar, 31] 1.10 [0.88, 1.38]
Multiple, United Kingdom of Great Britain and Northern Ireland [Fischbacher, 26] 0.86 [0.83, 0.89]
East Asia & Pacific, United Kingdom of Great Britain and Northern Ireland [Fischbacher, 26] 0.69 [0.57, 0.84]
East Asia & Pacific, United Kingdom of Great Britain and Northern Ireland [Fischbacher, 26] 0.72 [0.56, 0.92]
South Asia, United Kingdom of Great Britain and Northern Ireland [Fischbacher, 26] 0.44 [0.22, 0.90]
South Asia, United Kingdom of Great Britain and Northern Ireland [Fischbacher, 26] 0.63 [0.54, 0.73]
South Asia, United Kingdom of Great Britain and Northern Ireland [Fischbacher, 26] 0.88 [0.80, 0.96]
Europe & Central Asia, United Kingdom of Great Britain and Northern Ireland [Fischbacher, 26] 1.08 [1.03, 1.14]
Europe & Central Asia, United Kingdom of Great Britain and Northern Ireland [Fischbacher, 26] 1.00 [0.95, 1.06]
Europe & Central Asia, United Kingdom of Great Britain and Northern Ireland [Fischbacher, 26] 0.75 [0.74, 0.77]
Multiple, United Kingdom of Great Britain and Northern Ireland [Fischbacher, 26] 0.83 [0.80, 0.86]
East Asia & Pacific, United Kingdom of Great Britain and Northern Ireland [Fischbacher, 26] 0.86 [0.70, 1.06]
East Asia & Pacific, United Kingdom of Great Britain and Northern Ireland [Fischbacher, 26] 0.75 [0.59, 0.95]
South Asia, United Kingdom of Great Britain and Northern Ireland [Fischbacher, 26] 0.44 [0.16, 1.19]
South Asia, United Kingdom of Great Britain and Northern Ireland [Fischbacher, 26] 0.71 [0.59, 0.86]
South Asia, United Kingdom of Great Britain and Northern Ireland [Fischbacher, 26] 0.97 [0.88, 1.07]
Europe & Central Asia, United Kingdom of Great Britain and Northern Ireland [Fischbacher, 26] 1.02 [0.97, 1.07]
Europe & Central Asia, United Kingdom of Great Britain and Northern Ireland [Fischbacher, 26] 0.94 [0.89, 0.99]
Europe & Central Asia, United Kingdom of Great Britain and Northern Ireland [Fischbacher, 26] 0.82 [0.81, 0.83]
Europe & Central Asia, Germany [Deckert, 16] 0.96 [0.85, 1.09]
Europe & Central Asia, Germany [Deckert, 16] 0.85 [0.75, 0.97]
RE Model for Subgroup p = 0.00; I2 = 99.2%) 0.94 [0.89, 0.99]
RE Model 0.70 [0.65, 0.76]
0.14
0.22
0.37
0.61
1
1.65
2.72

Figure S4. Forest plots of SMRs for all-cause mortality by region of destination

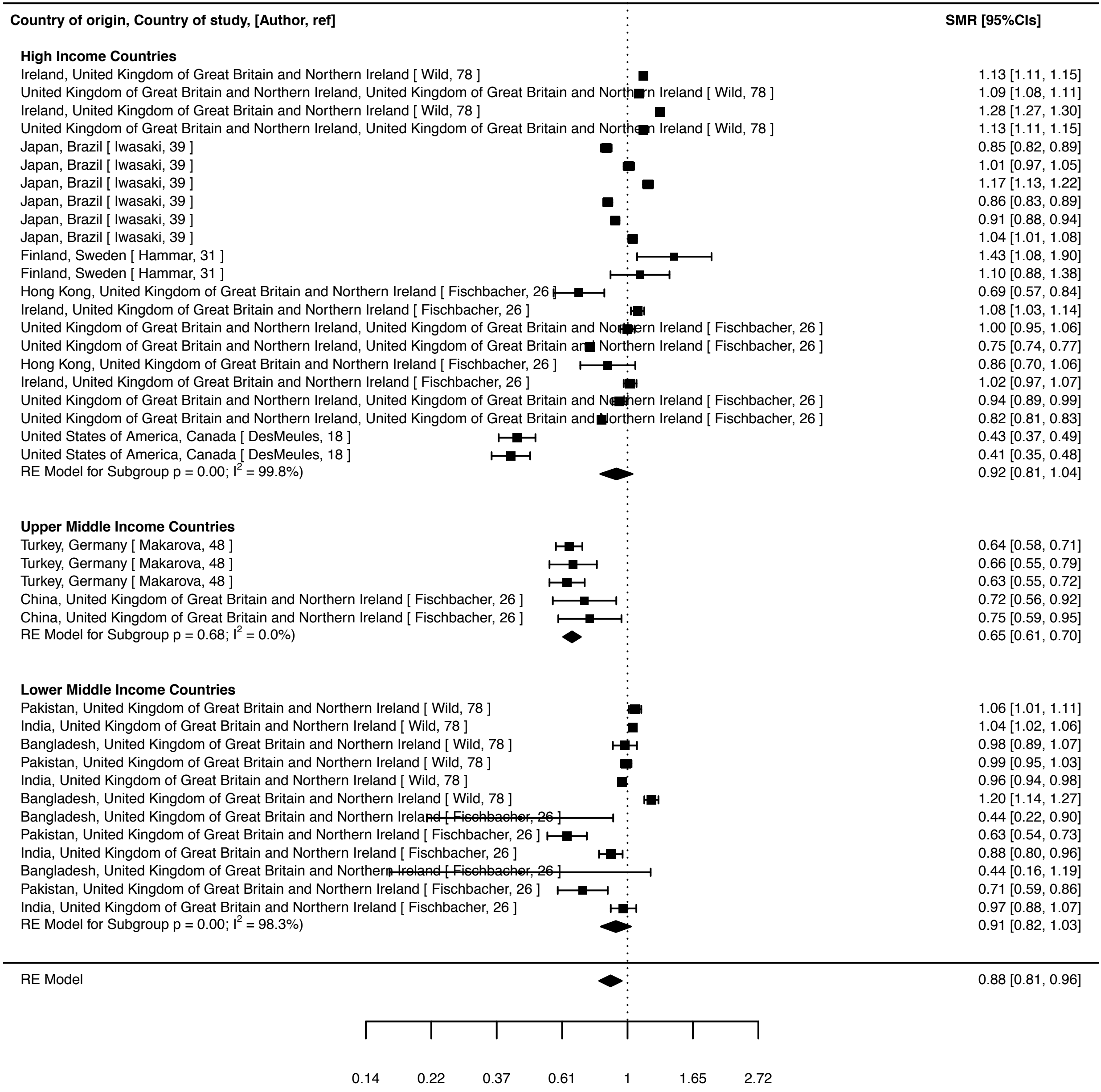
Country of origin, Country of study, [Author, ref]
SMR [95%CIs]
High Income Countries
Ireland, United Kingdom of Great Britain and Northern Ireland [Wild, 78] 1.13 [1.11, 1.15]
United Kingdom of Great Britain and Northern Ireland, United Kingdom of Great Britain and Northern Ireland [Wild, 78] 1.09 [1.08, 1.11]
Ireland, United Kingdom of Great Britain and Northern Ireland [Wild, 78] 1.28 [1.27, 1.30]
United Kingdom of Great Britain and Northern Ireland, United Kingdom of Great Britain and Northern Ireland [Wild, 78] 1.13 [1.11, 1.15]
Japan, Brazil [Iwasaki, 39] 0.85 [0.82, 0.89]
Japan, Brazil [Iwasaki, 39] 1.01 [0.97, 1.05]
Japan, Brazil [Iwasaki, 39] 1.17 [1.13, 1.22]
Japan, Brazil [Iwasaki, 39] 0.86 [0.83, 0.89]
Japan, Brazil [Iwasaki, 39] 0.91 [0.88, 0.94]
Japan, Brazil [Iwasaki, 39] 1.04 [1.01, 1.08]
Finland, Sweden [Hammar, 31] 1.43 [1.08, 1.90]
Finland, Sweden [Hammar, 31] 1.10 [0.88, 1.38]
Hong Kong, United Kingdom of Great Britain and Northern Ireland [Fischbacher, 26] 0.69 [0.57, 0.84]
Ireland, United Kingdom of Great Britain and Northern Ireland [Fischbacher, 26] 1.08 [1.03, 1.14]
United Kingdom of Great Britain and Northern Ireland, United Kingdom of Great Britain and Northern Ireland [Fischbacher, 26] 1.00 [0.95, 1.06]
United Kingdom of Great Britain and Northern Ireland, United Kingdom of Great Britain and Northern Ireland [Fischbacher, 26] 0.75 [0.74, 0.77]
Hong Kong, United Kingdom of Great Britain and Northern Ireland [Fischbacher, 26] 0.86 [0.70, 1.06]
Ireland, United Kingdom of Great Britain and Northern Ireland [Fischbacher, 26] 1.02 [0.97, 1.07]
United Kingdom of Great Britain and Northern Ireland, United Kingdom of Great Britain and Northern Ireland [Fischbacher, 26] 0.94 [0.89, 0.99]
United Kingdom of Great Britain and Northern Ireland, United Kingdom of Great Britain and Northern Ireland [Fischbacher, 26] 0.82 [0.81, 0.83]
United States of America, Canada [DesMeules, 18] 0.43 [0.37, 0.49]
United States of America, Canada [DesMeules, 18] 0.41 [0.35, 0.48]
RE Model for Subgroup p = 0.00; I2 = 99.8%) 0.92 [0.81, 1.04]
Upper Middle Income Countries
Turkey, Germany [Makarova, 48] 0.64 [0.58, 0.71]
Turkey, Germany [Makarova, 48] 0.66 [0.55, 0.79]
Turkey, Germany [Makarova, 48] 0.63 [0.55, 0.72]
China, United Kingdom of Great Britain and Northern Ireland [Fischbacher, 26] 0.72 [0.56, 0.92]
China, United Kingdom of Great Britain and Northern Ireland [Fischbacher, 26] 0.75 [0.59, 0.95]
RE Model for Subgroup p = 0.68; I2 = 0.0%) 0.65 [0.61, 0.70]
Lower Middle Income Countries
Pakistan, United Kingdom of Great Britain and Northern Ireland [Wild, 78] 1.06 [1.01, 1.11]
India, United Kingdom of Great Britain and Northern Ireland [Wild, 78] 1.04 [1.02, 1.06]
Bangladesh, United Kingdom of Great Britain and Northern Ireland [Wild, 78] 0.98 [0.89, 1.07]
Pakistan, United Kingdom of Great Britain and Northern Ireland [Wild, 78] 0.99 [0.95, 1.03]
India, United Kingdom of Great Britain and Northern Ireland [Wild, 78] 0.96 [0.94, 0.98]
Bangladesh, United Kingdom of Great Britain and Northern Ireland [Wild, 78] 1.20 [1.14, 1.27]
Bangladesh, United Kingdom of Great Britain and Northern Ireland [Fischbacher, 26] 0.44 [0.22, 0.90]
Pakistan, United Kingdom of Great Britain and Northern Ireland [Fischbacher, 26] 0.63 [0.54, 0.73]
India, United Kingdom of Great Britain and Northern Ireland [Fischbacher, 26] 0.88 [0.80, 0.96]
Bangladesh, United Kingdom of Great Britain and Northern Ireland [Fischbacher, 26] 0.44 [0.16, 1.19]
Pakistan, United Kingdom of Great Britain and Northern Ireland [Fischbacher, 26] 0.71 [0.59, 0.86]
India, United Kingdom of Great Britain and Northern Ireland [Fischbacher, 26] 0.97 [0.88, 1.07]
RE Model for Subgroup p = 0.00; I2 = 98.3%) 0.91 [0.82, 1.03]
RE Model 0.88 [0.81, 0.96]
0.14
0.22
0.37
0.61
1
1.65
2.72

Figure S5. Forest plots of SMRs for all-cause mortality by World Bank income group in country of origin

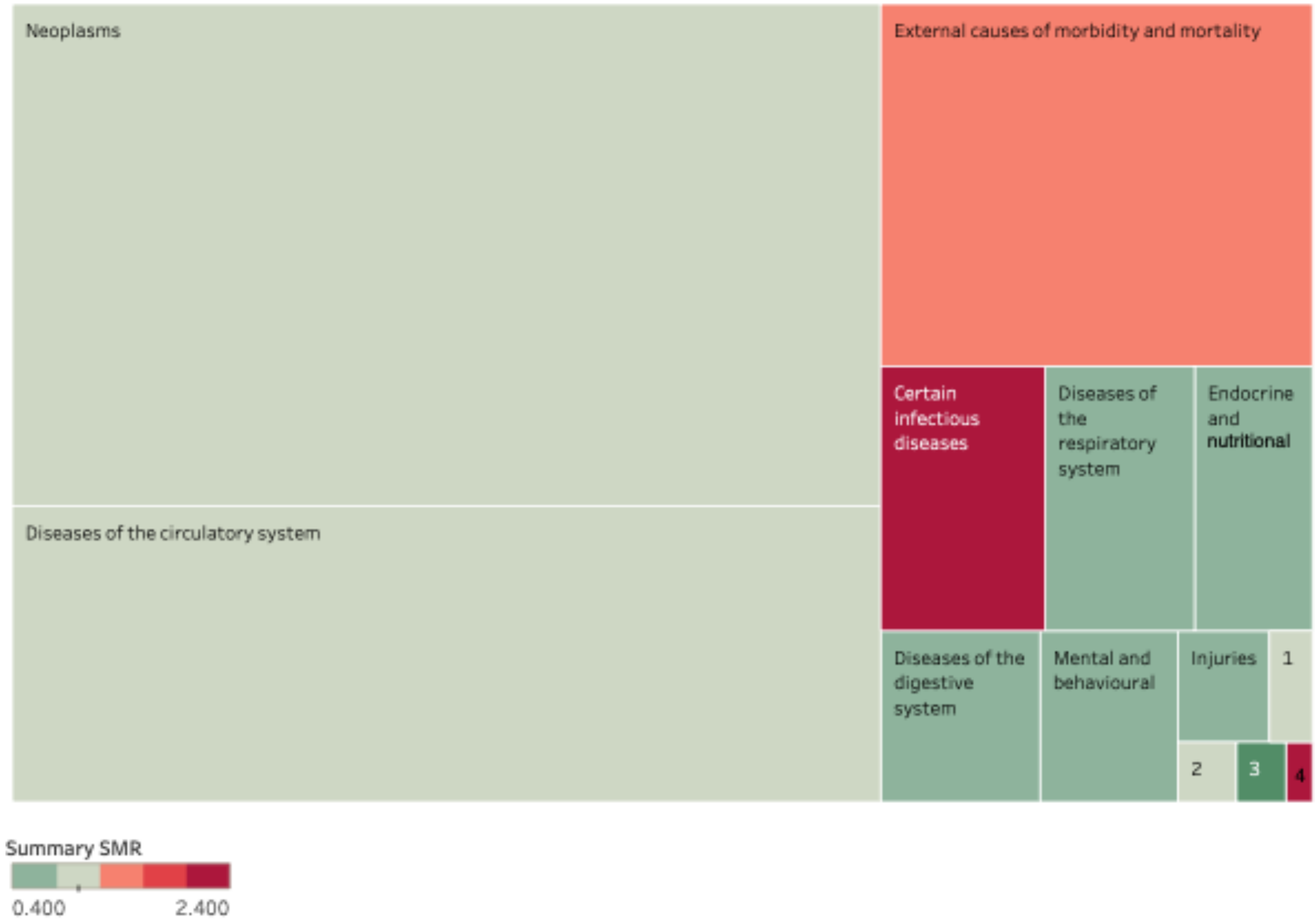

Figure S6. Treemap of mortality data points included in systematic review, by ICD-10 group and summary SMR.

1=Genitourinary;2=Musculoskeletal;3=Nervous;4=Blood.

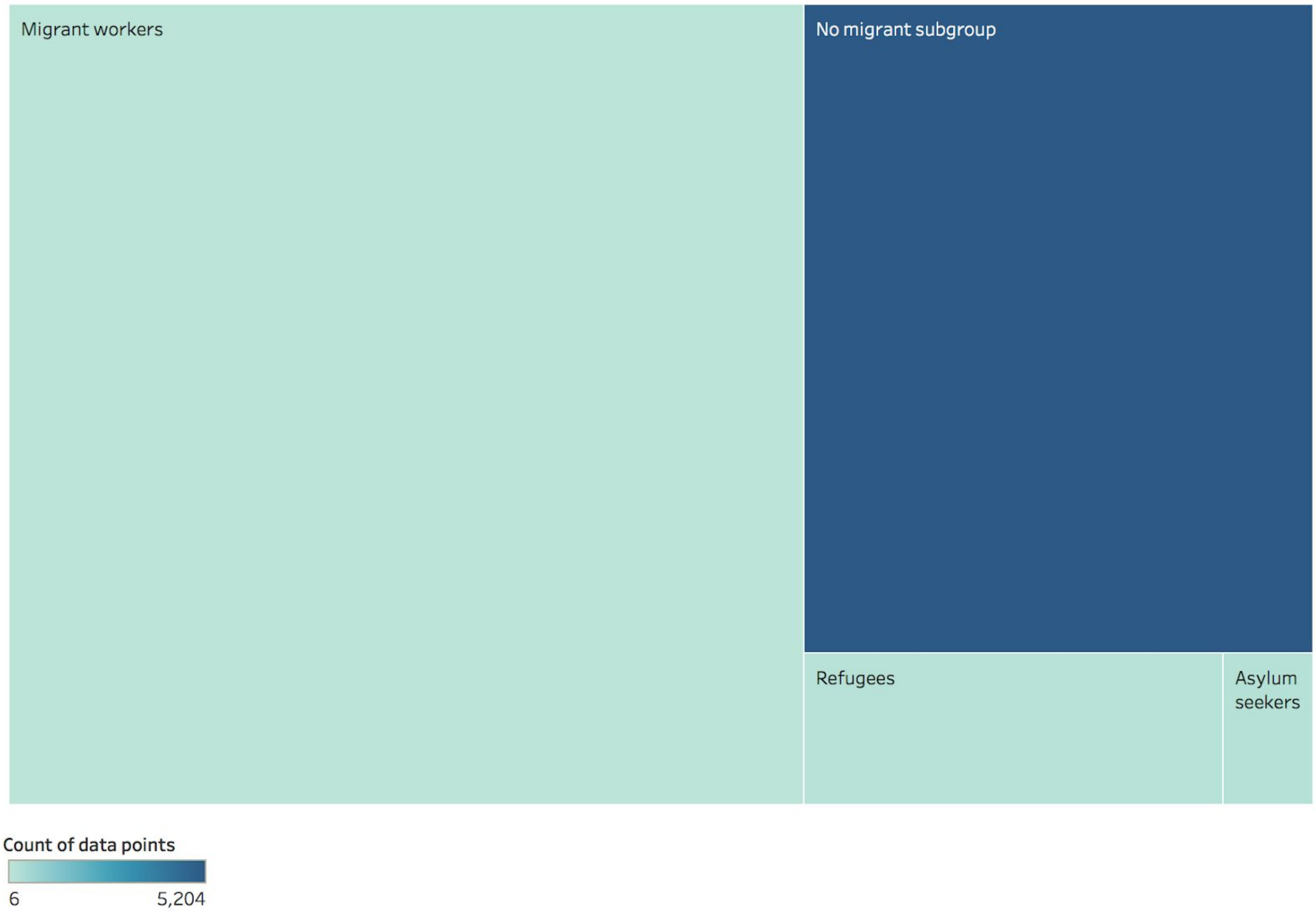

Figure S7. Treemap of mortality data points included in systematic review, by number of migrants in subgroup.

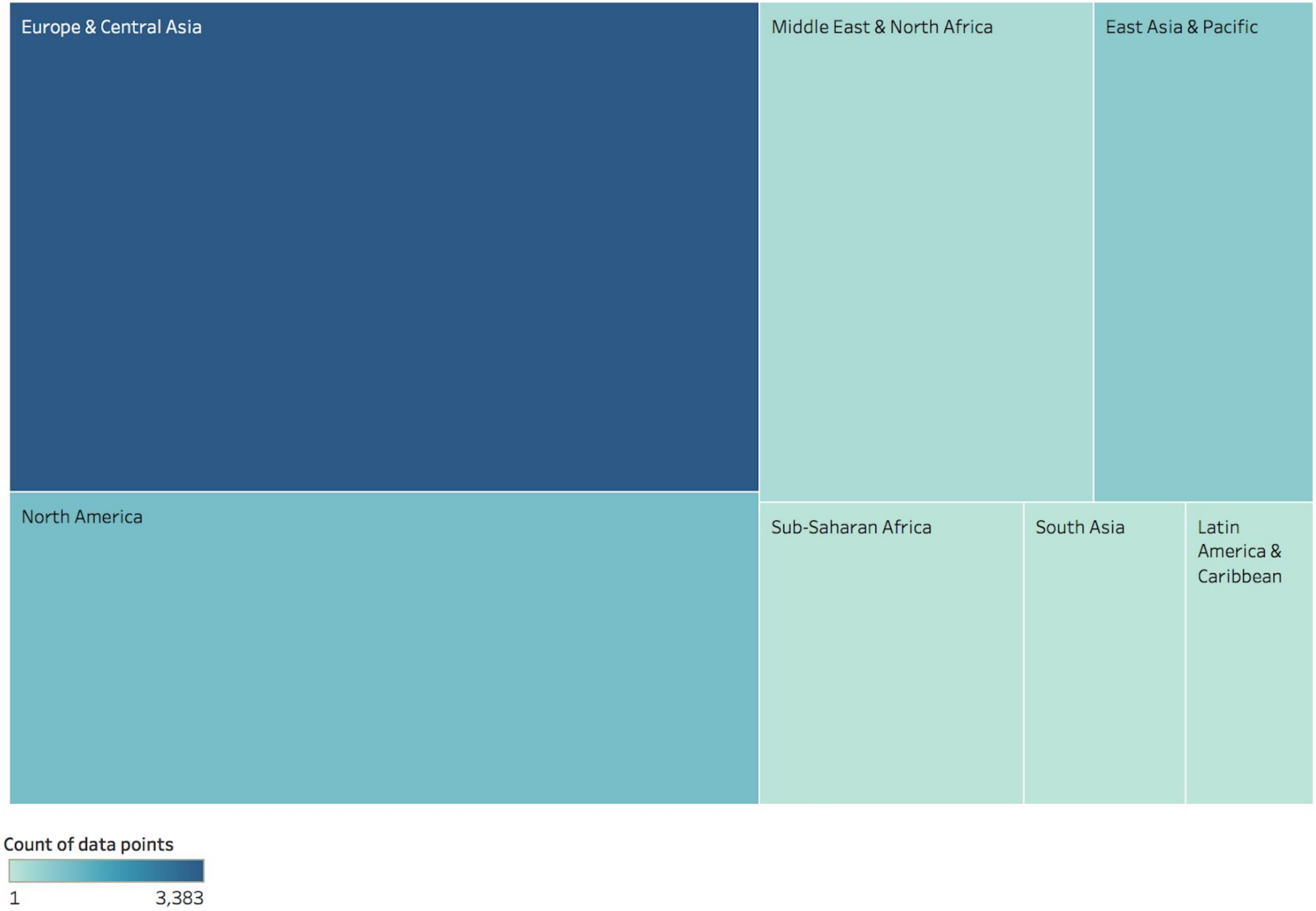

Figure S8. Treemap of mortality data points included in systematic review, by number of migrants in geographical region of destination.

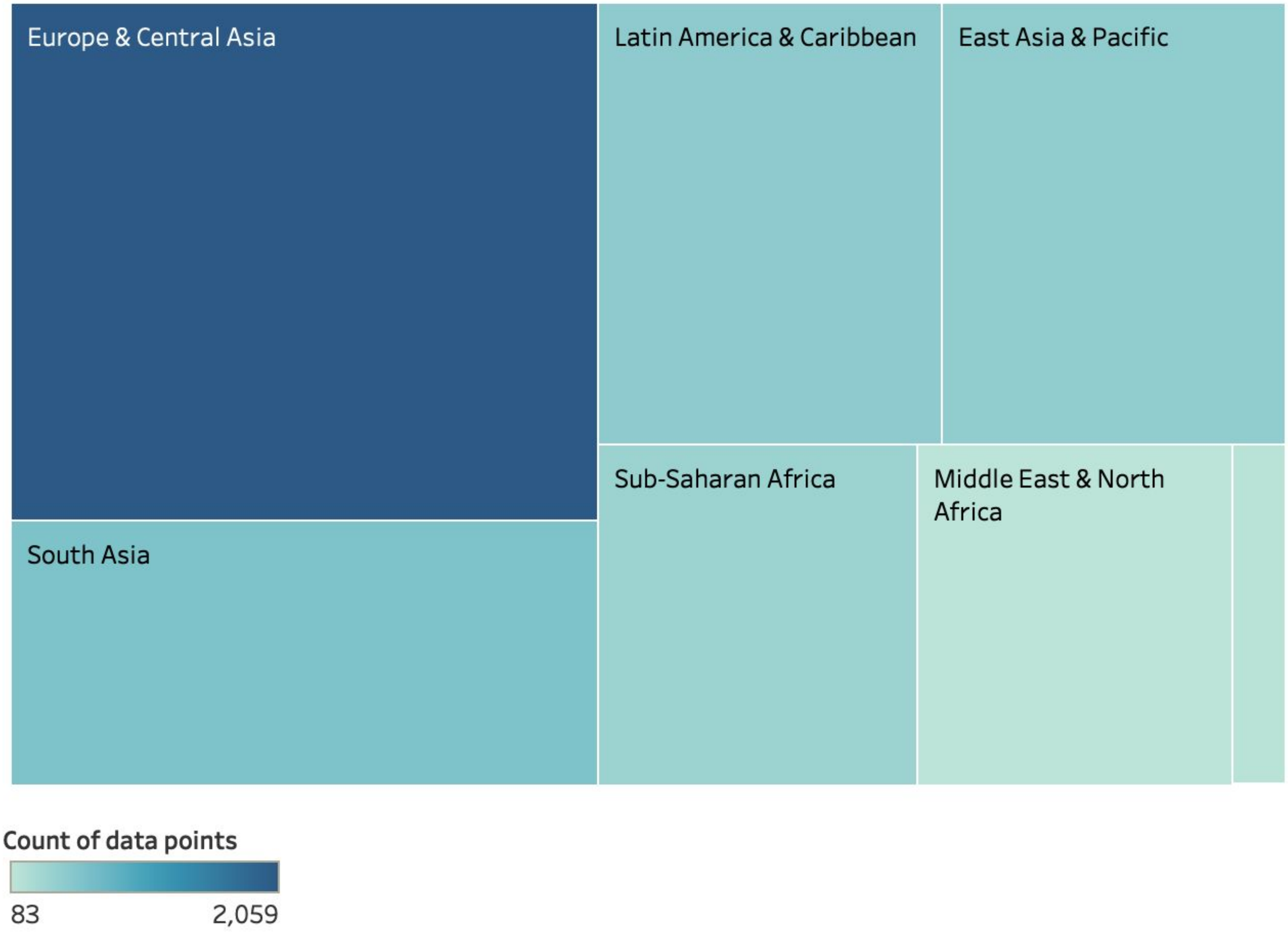

Figure S9. Treemap of mortality data points included in systematic review, by number of migrants in geographical region of origin.

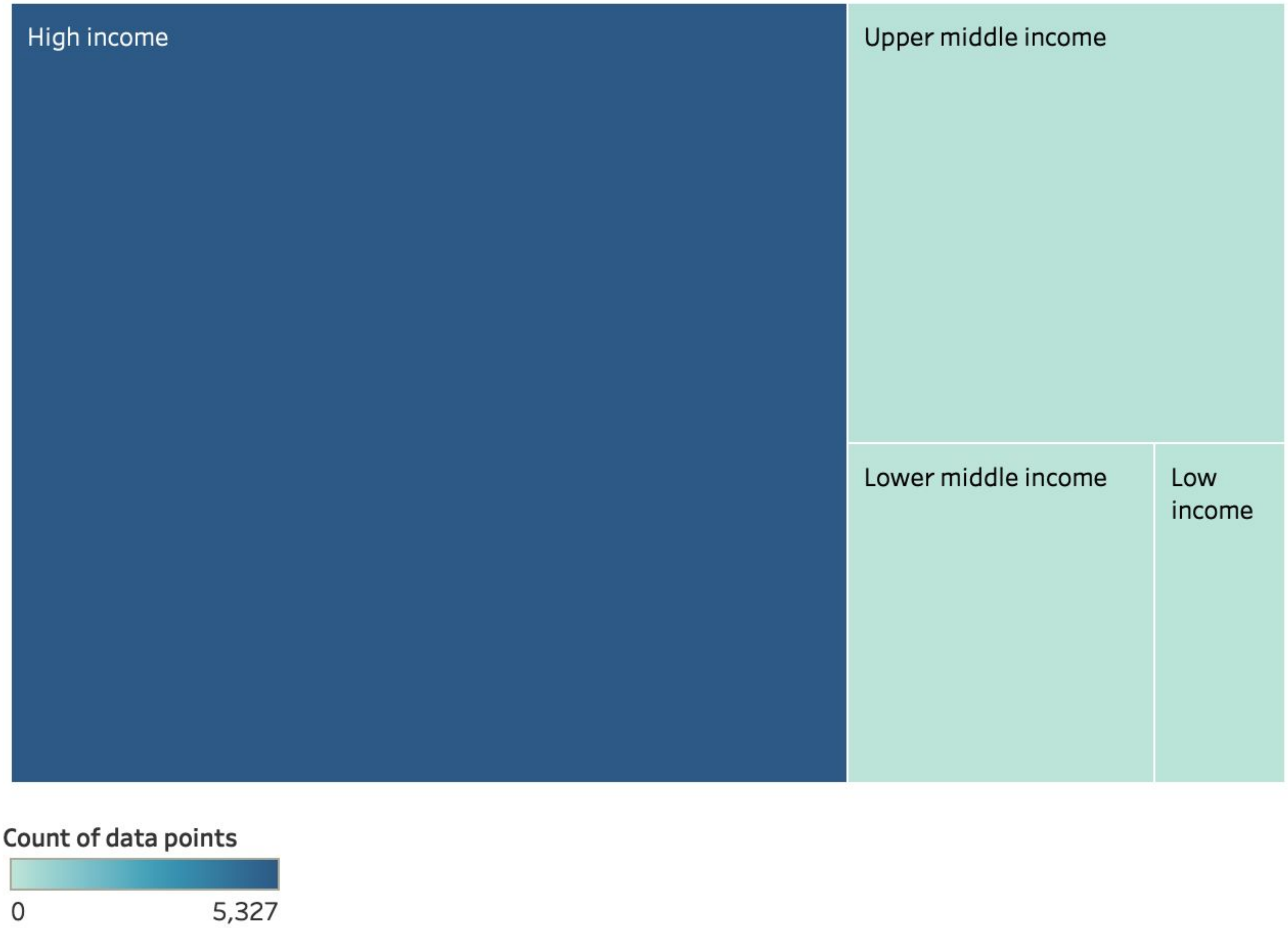

Figure S10. Treemap of mortality data points included in systematic review, by world bank income group in country of destination.

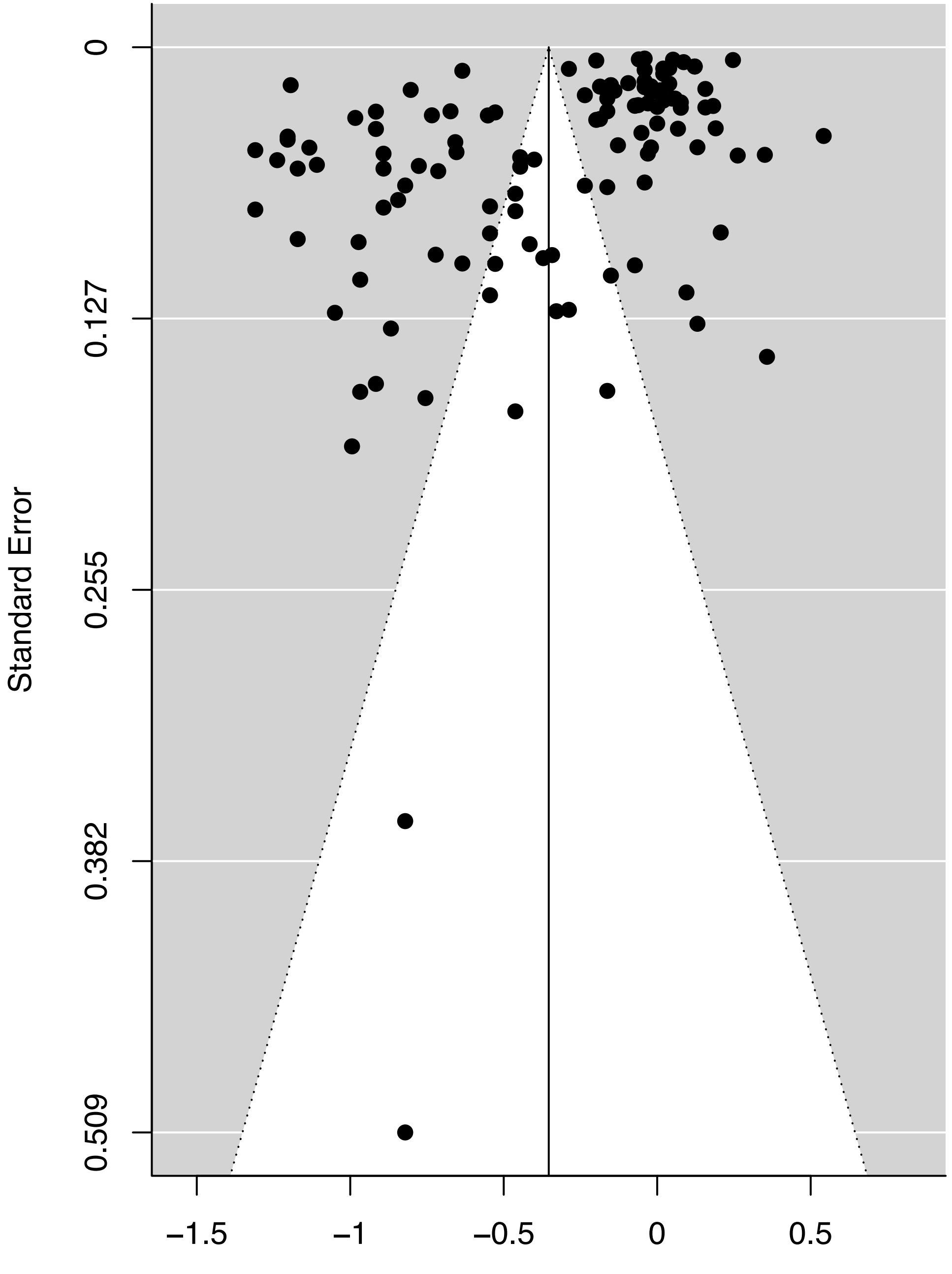
Standard Error
0
0.127
0.255
0.382
0.509
−1.5
−1
−0.5
0
0.5

Figure S11. Funnel plot of SMRs for all-cause mortality

Supplementary appendix - Tables

Table S1. Quality assessment: Cohort Studies

**Citation (author, date)**	**Selection**										
	**Representativeness of the exposed cohort/sample**				**Selection of the non-exposed cohort**			**Ascertainment of exposure**			
	Truly representative (one star)	Somewhat representative (one star)	Selected group (no star)	No description of the derivation of the cohort/sampling strategy (no star)	Drawn from the same community as the exposed cohort (one star)	Drawn from a different source (no star)	No description of the derivation of the non-exposed cohort (no star)	Secure record (one star)	Structured interview (one star)	Written self report (no star)	No description (no star)
Abdoli 2014[1]	1				1			1			
Aminisani 2012[2]	1				1			1			
Anikeeva 2012[3]	1				1			1			
Anikeeva 2015[4]	1				1			1			
CDC 2008[5]			0		1			1			
Antunes 2001[6]	1				1						0
Azziz-Baumgartner 2011[7]	1				1			1			
Becher 2007[8]	1				1			1			
Bhala 2010[9]	1				1			1			
Bhopal 2012[10]	1				1			1			
Bos 2007[11]	1				1			1			
CDC 2001[12]				0			0				0
Cha 2014[13]		1			1			1			
De Grande 2014[14]		1			1			1			
Deckert 2015[15]		1			1			1			
Deckert 2010[16]		1			1						0
DesMeules 2004[17]	1				1			1			
DesMeules 2005[18]	1				1			1			
El-Serag 2007[19]		1			1			1			
Elstad 2015[20]	1				1			1			
Eschbach 2006[21]	1				1			1			
Eschbach 2007[22]	1				1			1			
Esscher 2013[23]	1				1			1			
Feletto 2015[24]	1				1			1			
Fierro 2010[25]	1				1			1			
Fischbacher 2007[26]	1				1			1			
Gabbay 2014[27]	1				1			1			
Gadd 2006[28]	1				1			1			
Goosen 2011[29]	1				1			1			
Hajat 2010[30]		1			1			1			
Hammar 2002[31]		1			1			1			
Harding 2008[32]		1			1			1			
Hastings 2016[33]		1			1			1			

Herring 2010[34]	1				1			1			
Hjern 2002[35]		1			1			1			
Hjern 2006[36]	1				1			1			
Hollander 2012[37]	1				1			1			
Ikram 2016[38]	1				1			1			
Iwasaki 2008[39]	1				1			1			
Jung 2010[40]	1				1			1			
Kaucher 2017[41]	1				1			1			
Kibele 2008[42]		1			1			1			
Kohls 2015[43]		1			1			1			
Koppenaal 2003[44]		1			1			1			
Kyobutungi 2006[45]		1			1			1			
Kyobutungi 2006[46]		1			1			1			
Landman 2001[47]				0			0				0
Makarova 2016[48]	1				1			1			
Mangtani 2010[49]	1				1			1			
Moncho 2015[50]	1				1			1			
Mousavi 2012[51]	1				1			1			
Ng 2011[52]		1			1			1			
Omariba 2015[53]		1			1			1			
Omariba 2014 [54]		1			1			1			
Ott 2009[55]		1			1			1			
Ott 2008[56]		1			1			1			
Ott 2008[57]		1			1			1			
Patel 2004[58]		1			1						0
Pinheiro 2016[59]		1			1					0	
Rawshani 2015[60]		1			1			1			
Razum 2004[61]		1			1			1			
Regidor 2011[62]		1			1			1			
Regidor 2008[63]		1			1			1			
Regidor 2009[64]		1			1			1			
Reid 2016[65]		1			1			1			
Reiss 2013[66]		1					0	1			
Richard 2014[67]	1				1			1			
Ronellenfitsch 2006[68]		1			1			1			
Spallek 2012[69]	1				1			1			
Tarnutzer 2012[70]	1				1			1			
van Oostrum 2011[71]	1				1			1			
Vandenheede 2012[72]	1				1			1			
Vandenheede 2015[73]		1			1			1			
Vandenheede 2012[74]		1			1			1			
Verropoulou 2016[75]	1				1			1			
Wallace 2015[76]		1			1			1			
Westerling 2002[77]	1				1			1			

Wild 2007[78]	1				1			1			
Williamson 2009[79]		1			1			1			
Winkler 2009[80]		1					0	1			
Zeeb 2002[81]		1			1			1			

Citation (author, date)	Comparability		Outcome (one star per item)							
	**Comparability on the basis of the design or analysis controlled for confounders**		**Assessment of outcome**					**Was duration of follow up explicitly indicated?**		
	Study controls for relevant factors (e.g. age, sex) (one star)	Not comparable on the basis of study design or analysis (no star)	Independent blind assessment (one star)	Record linkage (one star)	Self report (no star)	No description (no star)	Other (no star)	Yes (one star)	No (no star)	Comment on the duration of follow-up
Abdoli 2014[1]	1			1				1		Follow up between 1961 and 2009. Total follow up is 412,386 million person-years.
Aminisani 2012[2]	1			1				1		Follow up between 1973 and 2008
Anikeeva 2012[3]	1			1				1		Follow up between 1981 and 2007
Anikeeva 2015[4]	1			1				1		Follow up between 1981 and 2007
CDC 2008[5]	0			1				1		Follow up between 1992 and 2006
Antunes 2001[6]	1			1				1		Follow up between 1994 and 1998
Azziz-Baumgartner 2011[7]	0			1				1		Person years indicated
Becher 2007[8]	1			1				1		Duration of follow up between 1st January 1990 and 31st December 2002; person-years given and mean years of follow up (7.4)
Bhala 2010[9]	1			1				1		Follow up between 2000 and 2004
Bhopal 2012[10]	1			1				1		Person years provided for each national cohort
Bos 2007[11]	1			1				1		Follow up between 1995 and 2000
CDC 2001[12]	0						0	1		Follow up between January and May 2001
Cha 2014[13]	1			1				1		Follow up between 2005 and 2007
De Grande 2014[14]	1			1				1		Follow up between 01/10/2001 01/01/2006
Deckert 2015[15]	1			1				1		Person years provided
Deckert 2010[16]	1					0		1		Follow up between 1980 and 2007
DesMeules 2004[17]		0		1				1		Follow up between 1980 and 1998
DesMeules 2005[18]	1			1				1		Follow up between 1980 and 1998. Person-years given.
El-Serag 2007[19]	1			1					0	Follow up between 1979 and 2001
Elstad 2015[20]	1			1				1		Follow up between 1993–1996 and 2008–2011
Eschbach 2006[21]	1			1				1		Follow up between 1999 and 2000
Eschbach 2007[19]	1			1				1		Follow up between 1999 and 2001
Esscher 2013[23]	1			1				1		Follow up between 1988 and 2007
Feletto 2015[24]	1			1				1		Follow up between 2004 and 2008
Fierro 2010[25]	0			1				1		Follow up during 2004
Fischbacher 2007[26]	1			1				1		Follow up between 1997 and 2003
Gabbay 2014[27]	1			1				1		Follow up between 1980 and 1996 (five years per cohort)
Gadd 2006[28]	1			1				1		Follow up between 1991 and 1998

Goosen 2011[29]	1			1				1		Follow up between 2002 and 2007
Hajat 2010[30]	1			1				1		Follow up between 1996 and 2004
Hammar 2002[31]	1			1				1		Follow up between 1976 and 1995
Harding 2008[32]	1			1				1		Follow up between 1998 and 2002
Hastings 2016[33]	1			1				1		Follow up between 2003 and 2011
Herring 2010[34]	1			1				1		Follow up between 1996 and 2005
Hjern 2002[35]		0		1				1		Follow up between 1986 and 1995
Hjern 2006[36]	1			1				1		Follow up between 1991 and 2000
Hollander 2012[37]	1			1				1		Follow up betwen 1998 and 2006
Ikram 2016[38]	1			1				1		Various (from different national registers)
Iwasaki 2008[39]	1			1				1		Follow up between 1979 and 2001
Jung 2010[40]	1			1				1		Follow up between 1990 and 2006
Kaucher 2017[41]	1			1				1		Follow up between 1990 and 2009
Kibele 2008[42]		0		1				1		Follow up between 1995 and 2004
Kohls 2015[43]	1			1				1		Follow up between 1970 and 2013
Koppenaal 2003[44]	1			1				1		Follow up between 1998 and 1999
Kyobutungi 2006[45]	1			1				1		Follow up between 1990 and 2001
Kyobutungi 2006[46]	1			1				1		Follow up between 1990 and 2002
Landman 2001[47]		0				0		1		Follow up between 1989 and 1992
Makarova 2016[48]	1			1				1		Follow up between 2004 and 2010
Mangtani 2010[49]	1			1				1		Follow up between 1993 and 2003
Moncho 2015[50]	1			1				1		Follow up between 1999 and 2008
Mousavi 2012[51]	1			1				1		Person years provided (total 12.7 million) and follow up between 1958 and 2008
Ng 2011[52]	1			1				1		Follow up between 1991 and 2001
Omariba 2015[53]	1			1				1		Follow up between 1991 and 2006
Omariba 2014 [54]	1			1				1		Follow up between 1991 and 2006
Ott 2009[55]	1			1				1		Follow up between 1990 and 2003
Ott 2008[56]	1			1				1		Person years provided; follow up between 1990 and 2002
Ott 2008[57]	1			1				1		Person years provided; follow up between 1990 and 2003 for Israeli cohort; follow up between 1990 and 2005 for German cohort
Patel 2004[58]	1			1				1		Follow up between 1993 and 2000
Pinheiro 2016[59]	1			1				1		Follow up between 2008 and 2012
Rawshani 2015[60]	1		1					1		150,541 person years of follow up; follow up between 2006 and 2012
Razum 2004[61]	1			1				1		Follow up between 1980 and 1997
Regidor 2011[62]	1			1				1		Person years provided
Regidor 2008[63]	1			1				1		Person years provided
Regidor 2009[64]	1			1				1		Person years provided
Reid 2016[65]	1			1				1		Person years provided
Reiss 2013[66]		0		1				1		Person years provided; Follow up between 1990 and 2005
Richard 2014[67]	1			1				1		Person years provided; Follow u between 1990 and 2008
Ronellenfitsch 2006[68]	1			1				1		Follow up between 1990 amd 2001
Spallek 2012[69]	1			1				1		Person years provided
Tarnutzer 2012[70]	1			1				1		Person years provided
van Oostrum 2011[71]	1			1				1		Follow up between 2002 and 2005
Vandenheede 2012[72]	1			1				1		Person years provided

Vandenheede 2015[73]	1			1				1		Person years provided
Vandenheede 2012[74]	1			1				1		Follow up between 2001 and 2005
Verropoulou 2016[75]	1			1				1		Follow up between 2010 and 2012
Wallace 2015[76]	0			1				1		Person years provided
Westerling 2002[77]	1			1				1		Follow up between 1986 and 1990
Wild 2007[78]	1			1				1		Follow up between 2001 and 2003
Williamson 2009[79]	1			1				1		Follow up between 1998 and 2002
Winkler 2009[80]	1			1				1		Follow up between 1990 and 2005
Zeeb 2002[81]	1			1				1		Follow up between 1980 and 1997

**Citation (author, date)**	**Outcome**						**Total score**		
	**Adequacy of follow-up cohorts**				**Statistical Test**				
	Complete follow up reported. All subjects accounted for (one star)	Subjects lost to follow-up are discussed or are unlikely to introduce bias (one star)	Subjects lost to follow-up are not discussed or may introduce bias (no star)	No reporting of subjects lost to follow-up (no star)	Are sufficient data presented to support the estimates or conclusions drawn? (measures of precision reported; denominators reported) (one star)	The statistical test is not appropriate, not described or incomplete (no star)	Numerator	Denominator (*all studies = 8*)	Percentage
Abdoli 2014[1]				0	1		7	8	0.875
Aminisani 2012[2]				0	1		7	8	0.875
Anikeeva 2012[3]				0		0	6	8	0.750
Anikeeva 2015[4]				0		0	6	8	0.750
CDC 2008[5]				0		0	4	8	0.500
Antunes 2001[6]				0		0	5	8	0.625
Azziz-Baumgartner 2011[7]				0	1		6	8	0.750
Becher 2007[8]		1			1		8	8	1.000
Bhala 2010[9]				0	1		7	8	0.875
Bhopal 2012[10]				0	1		7	8	0.875
Bos 2007[11]			0		1		7	8	0.875
CDC 2001[12]				0		0	1	8	0.125
Cha 2014[13]				0	1		7	8	0.875
De Grande 2014[14]		1			1		8	8	1.000
Deckert 2015[15]		1			1		8	8	1.000
Deckert 2010[16]				0		0	4	8	0.500
DesMeules 2004[17]			0			0	5	8	0.625
DesMeules 2005[18]		1			1		8	8	1.000
El-Serag 2007[19]				0		0	5	8	0.625
Elstad 2015[20]		1			1		8	8	1.000
Eschbach 2006[21]		1			1		8	8	1.000
Eschbach 2007[22]			0		1		7	8	0.875
Esscher 2013[23]		1			1		8	8	1.000
Feletto 2015[24]		1				0	7	8	0.875
Fierro 2010[25]			0			0	5	8	0.625
Fischbacher 2007[26]		1			1		8	8	1.000
Gabbay 2014[27]		1			1		8	8	1.000
Gadd 2006[28]		1			1		8	8	1.000

Goosen 2011[29]		1			1		8	8	1.000
Hajat 2010[30]			0		1		7	8	0.875
Hammar 2002[31]			0		1		7	8	0.875
Harding 2008[32]				0	1		7	8	0.875
Hastings 2016[33]				0	1		7	8	0.875
Herring 2010[34]		1				0	7	8	0.875
Hjern 2002[35]			0			0	5	8	0.625
Hjern 2006[36]		1				0	7	8	0.875
Hollander 2012[37]		1			1		8	8	1.000
Ikram 2016[38]		1			1		8	8	1.000
Iwasaki 2008[39]		1			1		8	8	1.000
Jung 2010[40]		1			1		8	8	1.000
Kaucher 2017[41]			0		1		7	8	0.875
Kibele 2008[42]			0			0	5	8	0.625
Kohls 2015[43]			0			0	6	8	0.750
Koppenaal 2003[44]			0		1		7	8	0.875
Kyobutungi 2006[45]			0		1		7	8	0.875
Kyobutungi 2006[46]			0		1		7	8	0.875
Landman 2001[47]				0		0	1	8	0.125
Makarova 2016[48]		1			1		8	8	1.000
Mangtani 2010[49]		1			1		8	8	1.000
Moncho 2015[50]				0	0		6	8	0.750
Mousavi 2012[51]				0	1		7	8	0.875
Ng 2011[52]				0		0	6	8	0.750
Omariba 2015[53]				0	1		7	8	0.875
Omariba 2014 [54]				0	1		7	8	0.875
Ott 2009[55]				0	1		7	8	0.875
Ott 2008[56]		1			1		8	8	1.000
Ott 2008[57]				0	1		7	8	0.875
Patel 2004[58]		1			1		7	8	0.875
Pinheiro 2016[59]				0	1		6	8	0.750
Rawshani 2015[60]				0	1		7	8	0.875
Razum 2004[61]			0		1		7	8	0.875
Regidor 2011[62]				0	1		7	8	0.875
Regidor 2008[63]				0	1		7	8	0.875
Regidor 2009[64]				0	1		7	8	0.875
Reid 2016[65]				0	1		7	8	0.875
Reiss 2013[66]		1			1		6	8	0.750
Richard 2014[67]				0	1		7	8	0.875
Ronellenfitsch 2006[68]		1			1		8	8	1.000
Spallek 2012[69]				0		0	6	8	0.750
Tarnutzer 2012[70]				0	1		7	8	0.875
van Oostrum 2011[71]				0	1		7	8	0.875
Vandenheede 2012[72]				0	1		7	8	0.875
Vandenheede 2015[73]		1			1		8	8	1.000
Vandenheede 2012[74]				0	1		7	8	0.875
Verropoulou 2016[75]				0	1		7	8	0.875
Wallace 2015[76]		1				0	6	8	0.750
Westerling 2002[77]				0	1		7	8	0.875

Wild 2007[78]		1			1		8	8	1.000
Williamson 2009[79]				0	1		7	8	0.875
Winkler 2009[80]		1			1		7	8	0.875
Zeeb 2002[81]				0	1		7	8	0.875

Table S2. Quality assessment: Cross-sectional Studies

Citation (author, date)	Selection								
	Representativeness of the exposed cohort/sample				Sample size		Non-respondents		
	Truly representative (one star)	Somewhat representative (one star)	Selected group (no star)	No description of the derivation of the cohort/sampling strategy (no star)	Justified and satisfactory (consider statistical power and transparency of reporting) (one star)	Not justified	Comparability between respondents and non-respondents characteristics is established, and the response rate is satisfactory (one star)	The response rate is unsatisfactory, or the comparability between respondents and non-respondents is unsatisfactory (no star)	No description of the response rate or the characteristics of the responders and the non-responders (no star)
Andresen 2014[82]				0	0				0
Bartlett 2002[83]	1				1				0
Blue 2011[84]		1			1				0
Cronin 2009[85]				0		0			0
Harding 2008[86]	1					0			0
Maynard 2012[87]		1				0			0
Muennig 2002[88]		1				0			0
Page 2007[89]	1					0			0
Rubia 2002[90]		1			1				0
Ruiz-Ramos 2013[91]	1				1		1		
Seeff 2003[92]		1			1				0
Shah 2009[93]		1			1				0
Singh 2006[94]		1			1				0
Singh 2013[95]		1			1				0
Tiagi 2015[96]		1							0

Citation (author, date)	Selection			Comparability	Outcome (one star per item)						Total Score		
	Ascertainment of the exposure (risk factor)			Comparability of cohorts on the basis of the design or analysis controlled for confounders	Assessment of outcome				Statistical test				
	Validated measurement tool (two stars)	Non-validated measurement tool, but the tool is available or described (one star)	No description of the measurement tool (no star)	The study controls for relevant factors (e.g. age and sex) (one star)	Independent blind assessment (one star)	Record linkage (one star)	self report (no star)	No description (no star)	Are sufficient data presented to support the estimates or conclusions drawn? (measures of precision reported; denominators reported) (one star)	The statistical test is not appropriate, not described or incomplete (no star)	**Numerator**	**Denominator (*all studies = 8*)**	**Percentage**
Andresen 2014[82]			0	0			0		1		1	8	0.125
Bartlett 2002[83]			0	0			0		1		3	8	0.375
Blue 2011[84]		1		1		1				0	5	8	0.625

Cronin 2009[85]			0	0				0		0	0	8	0.000
Harding 2008[86]			0	1		1			1		4	8	0.500
Maynard 2012[87]			0	1		1			1		4	8	0.500
Muennig 2002[88]	2			1		1				0	5	8	0.625
Page 2007[89]			0	0		1				0	2	8	0.250
Rubia 2002[90]		1		1		1			1		6	8	0.750
Ruiz-Ramos 2013[91]	2			1		1				0	7	8	0.875
Seeff 2003[92]		1		1		1			1		6	8	0.750
Shah 2009[93]			0	1		1			1		5	8	0.625
Singh 2006[94]		1		1		1				0	5	8	0.625
Singh 2013[95]		1		1		1				0	6	8	0.750
Tiagi 2015[96]			0	0		1				0	2	8	0.250

References

1 Abdoli G, Bottai M, Moradi T. Cancer mortality by country of birth, sex, and socioeconomic position in Sweden, 1961-2009. *PLoS One* 2014; **9**: e93174.

2 Aminisani N, Armstrong BK, Egger S, Canfell K. Impact of organised cervical screening on cervical cancer incidence and mortality in migrant women in Australia. *BMC Cancer* 2012; **12**: 491.

3 Anikeeva O, Bi P, Hiller JE, Ryan P, Roder D, Han G-S. Trends in cancer mortality rates among migrants in Australia: 1981-2007. *Cancer Epidemiol* 2012; **36**: e74–82.

4 Anikeeva O, Bi P, Hiller JE, Ryan P, Roder D, Han G-S. Trends in migrant mortality rates in Australia 1981-2007: a focus on the National Health Priority Areas other than cancer. *Ethn Health* 2015; **20**: 29–48.

5 Anonymous. Work-related injury deaths among hispanics--United States, 1992-2006. 2008.

6 Antunes JL, Waldman EA. The impact of AIDS, immigration and housing overcrowding on tuberculosis deaths in Sao Paulo, Brazil, 1994-1998. *Soc Sci Med* 2001; **52**: 1071–80.

7 Azziz-Baumgartner E, McKeown L, Melvin P, Dang Q, Reed J. Rates of femicide in women of different races, ethnicities, and places of birth: Massachusetts, 1993-2007. *J Interpers Violence* 2011; **26**: 1077–90.

8 Becher H., Razum O., Kyobutungi C., *et al.* Mortality of immigrants from the former Soviet Union: Results of a cohort study. 2007.

9 Bhala N, Fischbacher C, Bhopal R. Mortality for alcohol-related harm by country of birth in Scotland, 2000-2004: potential lessons for prevention. *Alcohol Alcohol* 2010; **45**: 552–6.

10 Bhopal RS, Rafnsson SB, Agyemang C, *et al.* Mortality from circulatory diseases by specific country of birth across six European countries: test of concept. *Eur J Public Health* 2012; **22**: 353–9.

11 Bos V, Kunst AE, Garssen J, Mackenbach JP. Duration of residence was not consistently related to immigrant mortality. *J Clin Epidemiol* 2007; **60**: 585–92.

12 Centers for Disease Control and Prevention (CDC). Surveillance of mortality during a refugee crisis--Guinea, January-May 2001. *MMWR Morb Mortal Wkly Rep* 2001; **50**: 1029–32.

13 Cha S, Cho Y. Fatal and non-fatal occupational injuries and diseases among migrant and native workers in South Korea. *Am J Ind Med* 2014; **57**: 1043–52.

14 De Grande H, Vandenheede H, Gadeyne S, Deboosere P. Health status and mortality rates of adolescents and young adults in the Brussels-Capital Region: differences according to region of origin and migration history. *Ethn Health* 2014; **19**: 122–43.

15 Deckert A, Winkler V, Meisinger C, Heier M, Becher H. Suicide and external mortality pattern in a cohort of migrants from the former Soviet Union to Germany. *J Psychiatr Res* 2015; **63**: 36–42.

16 Deckert A, Winkler V, Paltiel A, Razum O, Becher H. Time trends in cardiovascular disease mortality in Russia and Germany from 1980 to 2007 - are there migration effects? *BMC Public Health* 2010; **10**: 488.

17 DesMeules M, Gold J, Kazanjian A, *et al.* New approaches to immigrant health assessment. *Canadian journal of public health = Revue canadienne de sante publique* 2004; **95**: I22–6.

18 DesMeules M, Gold J, McDermott S, *et al.* Disparities in mortality patterns among Canadian immigrants and refugees, 1980-1998: results of a national cohort study. *J Immigr Health* 2005; **7**: 221–32.

19 El-Serag HB, Lau M, Eschbach K, Davila J, Goodwin J. Epidemiology of hepatocellular carcinoma in Hispanics in the United States. *Arch Intern Med* 2007; **167**: 1983–9.

20 Elstad JI, Overbye E, Dahl E. Prospective register-based study of the impact of immigration on educational inequalities in mortality in Norway. *BMC Public Health* 2015; **15**: 364.

21 Eschbach K, Kuo Y-F, Goodwin JS. Ascertainment of Hispanic ethnicity on California death certificates: implications for the explanation of the Hispanic mortality advantage. *Am J Public Health* 2006; **96**: 2209–15.

22 Eschbach K, Stimpson JP, Kuo Y-F, Goodwin JS. Mortality of foreign-born and US-born Hispanic adults at younger ages: a reexamination of recent patterns. *Am J Public Health* 2007; **97**: 1297–304.

23 Esscher A, Haglund B, Hogberg U, Essen B. Excess mortality in women of reproductive age from low-income countries: a Swedish national register study. *Eur J Public Health* 2013; **23**: 274–9.

24 Feletto E, Sitas F. Quantifying disparities in cancer incidence and mortality of Australian residents of New South Wales (NSW) by place of birth: an ecological study. *BMC Public Health* 2015; **15**: 823.

25 Fierro I, Yanez JL, Alvarez FJ. Differences in alcohol-related mortality between foreign-born and native-born Spaniards. *Int J Drug Policy* 2010; **21**: 240–3.

26 Fischbacher CM, Steiner M, Bhopal R, *et al.* Variations in all cause and cardiovascular mortality by country of birth in Scotland, 1997-2003. *Scott Med J* 2007; **52**: 5–10.

27 Gabbay U, Leshukovits Y, Sadetzki S. Immigrants' mortality patterns in the short- and long-term point toward origin-related diversities: the Israeli experience. *J Immigr Minor Health* 2014; **16**: 35–43.

28 Gadd M, Johansson S-E, Sundquist J, Wandell P. Are there differences in all-cause and coronary heart disease mortality between immigrants in Sweden and in their country of birth? A follow-up study of total populations. *BMC Public Health* 2006; **6**: 102.

29 Goosen S, Kunst AE, Stronks K, van Oostrum IEA, Uitenbroek DG, Kerkhof AJFM. Suicide death and hospital-treated suicidal behaviour in asylum seekers in the Netherlands: a national registry-based study. *BMC Public Health* 2011; **11**: 484.

30 Hajat A, Blakely T, Dayal S, Jatrana S. Do New Zealand's immigrants have a mortality advantage? Evidence from the New Zealand Census-Mortality Study. *Ethn Health* 2010; **15**: 531–47.

31 Hammar N, Kaprio J, Hagstrom U, Alfredsson L, Koskenvuo M, Hammar T. Migration and mortality: a 20 year follow up of Finnish twin pairs with migrant co-twins in Sweden. *J Epidemiol Community Health* 2002; **56**: 362–6.

32 Harding S, Teyhan A, Rosato M, Santana P. All cause and cardiovascular mortality in African migrants living in Portugal: evidence of large social inequalities. *Eur J Cardiovasc Prev Rehabil* 2008; **15**: 670–6.

33 Hastings K.G., Eggleston K., Boothroyd D., *et al.* Mortality outcomes for Chinese and Japanese immigrants in the USA and countries of origin (Hong Kong, Japan): A comparative analysis using national mortality records from 2003 to 2011. 2016. http://bmjopen.bmj.com/content/early/by/section.

34 Herring AA, Bonilla-Carrion RE, Borland RM, Hill KH. Differential mortality patterns between Nicaraguan immigrants and native-born residents of Costa Rica. *J Immigr Minor Health* 2010; **12**: 33–42.

35 Hjern A, Lindblad F, Vinnerljung B. Suicide, psychiatric illness, and social maladjustment in intercountry adoptees in Sweden: a cohort study. *Lancet* 2002; **360**: 443–8.

36 Hjern F., Johansson C., Mellgren A., Baxter N.N., Hjern A. Diverticular disease and migration - The influence of acculturation to a Western lifestyle on diverticular disease. 2006.

37 Hollander A-C, Bruce D, Ekberg J, Burstrom B, Borrell C, Ekblad S. Longitudinal study of mortality among refugees in Sweden. *Int J Epidemiol* 2012; **41**: 1153–61.

38 Ikram U.Z., Mackenbach J.P., Harding S., *et al.* All-cause and cause-specific mortality of different migrant populations in Europe. 2016. http://www.wkap.nl/journalhome.htm/0393-2990.

39 Iwasaki M, Mameri CP, Hamada GS, Tsugane S. Secular trends in cancer mortality among Japanese immigrants in the state of Sao Paulo, Brazil, 1979-2001. *Eur J Cancer Prev* 2008; **17**: 1–8.

40 Jung RS, Bennion JR, Sorvillo F, Bellomy A. Trends in tuberculosis mortality in the United States, 1990-2006: a population-based case-control study. *Public Health Rep* 2010; **125**: 389–97.

41 Kaucher S, Leier V, Deckert A, *et al.* Time trends of cause-specific mortality among resettlers in Germany, 1990 through 2009. *Eur J Epidemiol* 2017; **32**: 289–98.

42 Kibele E, Scholz R, Shkolnikov VM. Low migrant mortality in Germany for men aged 65 and older: fact or artifact? *Eur J Epidemiol* 2008; **23**: 389–93.

43 Kohls M. [Mortality risks of migrants: Analysis of the healthy-migrant-effect after the 2011 German Census]. *Bundesgesundheitsblatt Gesundheitsforschung Gesundheitsschutz* 2015; **58**: 519–26.

44 Koppenaal H., Bos C.A., Broer J. High mortality due to infectious diseases and unnatural causes of death among asylum seekers in the Netherlands, 1998-1999. 2003.

45 Kyobutungi C, Ronellenfitsch U, Razum O, Becher H. Mortality from external causes among ethnic German immigrants from former Soviet Union countries, in Germany. *Eur J Public Health* 2006; **16**: 376–82.

46 Kyobutungi C, Ronellenfitsch U, Razum O, Becher H. Mortality from cancer among ethnic German immigrants from the Former Soviet Union, in Germany. *Eur J Cancer* 2006; **42**: 2577–84.

47 Landman J, Cruickshank JK. A review of ethnicity, health and nutrition-related diseases in relation to migration in the United Kingdom. *Public Health Nutr* 2001; **4**: 647–57.

48 Makarova N, Brand T, Brunings-Kuppe C, Pohlabeln H, Luttmann S. Comparative analysis of premature mortality among urban immigrants in Bremen, Germany: a retrospective register-based linkage study. *BMJ Open* 2016; **6**: e007875.

49 Mangtani P, Maringe C, Rachet B, Coleman MP, dos Santos Silva I. Cancer mortality in ethnic South Asian migrants in England and Wales (1993-2003): patterns in the overall population and in first and subsequent generations. *Br J Cancer* 2010; **102**: 1438–43.

50 Moncho J, Pereyra-Zamora P, Nolasco A, Tamayo-Fonseca N, Melchor I, Macia L. Trends and Disparities in Mortality Among Spanish-Born and Foreign-Born Populations Residing in Spain, 1999-2008. *J Immigr Minor Health* 2015; **17**: 1374–84.

51 Mousavi SM, Sundquist K, Hemminki K. Morbidity and mortality in gynecological cancers among first- and second-generation immigrants in Sweden. *International journal of cancer* 2012; **131**: 497–504.

52 Ng E. The healthy immigrant effect and mortality rates. *Health Rep* 2011; **22**: 25–9.

53 Omariba DWR. Immigration, ethnicity, and avoidable mortality in Canada, 1991-2006. *Ethn Health* 2015; **20**: 409–36.

54 Omariba DWR, Ng E, Vissandjee B. Differences between immigrants at various durations of residence and host population in all-cause mortality, Canada 1991-2006. *Popul Stud* 2014; **68**: 339–57.

55 Ott JJ, Paltiel AM, Becher H. Noncommunicable disease mortality and life expectancy in immigrants to Israel from the former Soviet Union: country of origin compared with host country. *Bull World Health Organ* 2009; **87**: 20–9.

56 Ott JJ, Winkler V, Kyobutungi C, Laki J, Becher H. Effects of residential changes and time patterns on external-cause mortality in migrants: results of a German cohort study. *Scand J Public Health* 2008; **36**: 524–31.

57 Ott JJ, Paltiel AM, Winkler V, Becher H. Chronic disease mortality associated with infectious agents: a comparative cohort study of migrants from the

Former Soviet Union in Israel and Germany. *BMC Public Health* 2008; **8**: 110.

58 Patel KV, Eschbach K, Ray LA, Markides KS. Evaluation of mortality data for older Mexican Americans: implications for the Hispanic paradox. *Am J Epidemiol* 2004; **159**: 707–15.

59 Pinheiro PS, Callahan KE, Ragin C, Hage RW, Hylton T, Kobetz EN. Black Heterogeneity in Cancer Mortality: US-Blacks, Haitians, and Jamaicans. *Cancer Control* 2016; **23**: 347–58.

60 Rawshani A, Svensson A-M, Rosengren A, Eliasson B, Gudbjornsdottir S. Impact of Socioeconomic Status on Cardiovascular Disease and Mortality in 24,947 Individuals With Type 1 Diabetes. *Diabetes Care* 2015; **38**: 1518–27.

61 Razum O., Zeeb H. Suicide mortality among Turks in Germany. 2004.

62 Regidor E, Astasio P, Ortega P, Martinez D, Calle ME, de la Fuente L. Healthy and unhealthy migrant effect on the mortality of immigrants from wealthy countries residing in Spain. *Eur J Epidemiol* 2011; **26**: 265–73.

63 Regidor E, de La Fuente L, Martinez D, Calle ME, Dominguez V. Heterogeneity in cause-specific mortality according to birthplace in immigrant men residing in Madrid, Spain. *Ann Epidemiol* 2008; **18**: 605–13.

64 Regidor E, Astasio P, Calle ME, Martinez D, Ortega P, Dominguez V. The association between birthplace in different regions of the world and cardiovascular mortality among residents of Spain. *Eur J Epidemiol* 2009; **24**: 503–12.

65 Reid A, Peters S, Felipe N, Lenguerrand E, Harding S. The impact of migration on deaths and hospital admissions from work-related injuries in Australia. *Aust N Z J Public Health* 2016; **40**: 49–54.

66 Reiss K, Berger U, Winkler V, Voigtlander S, Becher H, Razum O. Assessing the effect of regional deprivation on mortality avoiding compositional bias: a natural experiment. *J Epidemiol Community Health* 2013; **67**: 213–8.

67 Richard A, Faeh D, Rohrmann S, Braun J, Tarnutzer S, Bopp M. Italianity is associated with lower risk of prostate cancer mortality in Switzerland. *Cancer Causes Control* 2014; **25**: 1523–9.

68 Ronellenfitsch U, Kyobutungi C, Becher H, Razum O. All-cause and cardiovascular mortality among ethnic German immigrants from the Former Soviet Union: a cohort study. *BMC Public Health* 2006; **6**: 16.

69 Spallek J, Arnold M, Razum O, *et al.* Cancer mortality patterns among Turkish immigrants in four European countries and in Turkey. *Eur J Epidemiol* 2012; **27**: 915–21.

70 Tarnutzer S, Bopp M, SNC Study Group. Healthy migrants but unhealthy offspring? A retrospective cohort study among Italians in Switzerland. *BMC Public Health* 2012; **12**: 1104.

71 van Oostrum IEA, Goosen S, Uitenbroek DG, Koppenaal H, Stronks K. Mortality and causes of death among asylum seekers in the Netherlands,

2002-2005. *J Epidemiol Community Health* 2011; **65**: 376–83.

72 Vandenheede H, Deboosere P, Stirbu I, *et al.* Migrant mortality from diabetes mellitus across Europe: the importance of socio-economic change. *Eur J Epidemiol* 2012; **27**: 109–17.

73 Vandenheede H, Willaert D, De Grande H, Simoens S, Vanroelen C. Mortality in adult immigrants in the 2000s in Belgium: a test of the 'healthy-migrant' and the 'migration-as-rapid-health-transition' hypotheses. *Trop Med Int Health* 2015; **20**: 1832–45.

74 Vandenheede H, Deboosere P, Gadeyne S, De Spiegelaere M. The associations between nationality, fertility history and diabetes-related mortality: a retrospective cohort study in the Brussels-Capital Region (2001-2005). *J Public Health* 2012; **34**: 100–7.

75 Verropoulou G, Tsimbos C. Mortality by Cause of Death Among Immigrants and Natives in a South European Country: The Case of Greece, 2011. *J Immigr Minor Health* 2016; **18**: 337–44.

76 Wallace M, Kulu H. Mortality among immigrants in England and Wales by major causes of death, 1971-2012: A longitudinal analysis of register-based data. *Soc Sci Med* 2015; **147**: 209–21.

77 Westerling R, Rosen M. 'Avoidable' mortality among immigrants in Sweden. *Eur J Public Health* 2002; **12**: 279–86.

78 Wild SH, Fischbacher C, Brock A, Griffiths C, Bhopal R. Mortality from all causes and circulatory disease by country of birth in England and Wales 2001-2003. *J Public Health* 2007; **29**: 191–8.

79 Williamson LM, Rosato M, Teyhan A, Santana P, Harding S. AIDS mortality in African migrants living in Portugal: evidence of large social inequalities. *Sex Transm Infect* 2009; **85**: 427–31.

80 Winkler V, Ott JJ, Holleczek B, Stegmaier C, Becher H. Cancer profile of migrants from the Former Soviet Union in Germany: incidence and mortality. *Cancer Causes Control* 2009; **20**: 1873–9.

81 Zeeb H, Razum O, Blettner M, Stegmaier C. Transition in cancer patterns among Turks residing in Germany. *Eur J Cancer* 2002; **38**: 705–11.

82 Andresen E, Bilukha OO, Menkir Z, *et al.* Notes from the field: malnutrition and elevated mortality among refugees from South Sudan - Ethiopia, June-July 2014. *MMWR Morb Mortal Wkly Rep* 2014; **63**: 700–1.

83 Bartlett LA, Jamieson DJ, Kahn T, Sultana M, Wilson HG, Duerr A. Maternal mortality among Afghan refugees in Pakistan, 1999-2000. *Lancet* 2002; **359**: 643–9.

84 Blue L, Fenelon A. Explaining low mortality among US immigrants relative to native-born Americans: the role of smoking. *Int J Epidemiol* 2011; **40**: 786–93.

85 Cronin AA, Shrestha D, Spiegel P, Gore F, Hering H. Quantifying the burden of disease associated with inadequate provision of water and sanitation in selected sub-Saharan refugee camps. *J Water Health* 2009; **7**: 557–68.

86 Harding S, Rosato M, Teyhan A. Trends for coronary heart disease and stroke mortality among migrants in England and Wales, 1979-2003: slow declines notable for some groups. *Heart* 2008; **94**: 463–70.

87 Maynard MJ, Rosato M, Teyhan A, Harding S. Trends in suicide among migrants in England and Wales 1979-2003. *Ethn Health* 2012; **17**: 135–40.

88 Muennig P, Fahs MC. Health status and hospital utilization of recent immigrants to New York City. *Prev Med* 2002; **35**: 225–31.

89 Page A, Begg S, Taylor R, Lopez AD. Global comparative assessments of life expectancy: the impact of migration with reference to Australia. *Bull World Health Organ* 2007; **85**: 474–81.

90 Rubia M, Marcos I, Muennig PA. Increased risk of heart disease and stroke among foreign-born females residing in the United States. *Am J Prev Med* 2002; **22**: 30–5.

91 Ruiz-Ramos M., Juarez S. All-cause and cause-specific mortality in the immigrant and native-born populations in Andalusia (Spain). 2013.

92 Seeff LC, McKenna MT. Cervical cancer mortality among foreign-born women living in the United States, 1985 to 1996. *Cancer Detect Prev* 2003; **27**: 203–8.

93 Shah A, Lindesay J, Dennis M. Comparison of elderly suicide rates among migrants in England and Wales with their country of origin. *Int J Geriatr Psychiatry* 2009; **24**: 292–9.

94 Singh GK, Hiatt RA. Trends and disparities in socioeconomic and behavioural characteristics, life expectancy, and cause-specific mortality of native-born and foreign-born populations in the United States, 1979-2003. *Int J Epidemiol* 2006; **35**: 903–19.

95 Singh GK, Rodriguez-Lainz A, Kogan MD. Immigrant health inequalities in the United States: use of eight major national data systems. *ScientificWorldJournal* 2013; **2013**: 512313.

96 Tiagi R. Are immigrants in Canada over-represented in riskier jobs relative to Canadian-born labor market participants? *Am J Ind Med* 2015; **58**: 933–42.

Supplementary appendix - Data and scripts

All data, the data extraction form and R Scripts used in this analysis are available online at the UCL Discovery service via the following DOI:

https://doi.org/10.14324/000.ds.10062606
